# The Role of Peptide Hormones in Insect Lipid Metabolism

**DOI:** 10.3389/fphys.2020.00434

**Published:** 2020-05-07

**Authors:** Umut Toprak

**Affiliations:** Molecular Entomology Lab., Department of Plant Protection Ankara, Faculty of Agriculture, Ankara University, Ankara, Turkey

**Keywords:** peptide hormones, neuropeptides, lipid metabolism, lipolysis, lipogenesis, adipokinetic hormone, insulin

## Abstract

Lipids are the primary storage molecules and an essential source of energy in insects during reproduction, prolonged periods of flight, starvation, and diapause. The coordination center for insect lipid metabolism is the fat body, which is analogous to the vertebrate adipose tissue and liver. The fat body is primarily composed of adipocytes, which accumulate triacylglycerols in intracellular lipid droplets. Genomics and proteomics, together with functional analyses, such as RNA interference and CRISPR/Cas9-targeted genome editing, identified various genes involved in lipid metabolism and elucidated their functions. However, the endocrine control of insect lipid metabolism, in particular the roles of peptide hormones in lipogenesis and lipolysis are relatively less-known topics. In the current review, the neuropeptides that directly or indirectly affect insect lipid metabolism are introduced. The primary lipolytic and lipogenic peptide hormones are adipokinetic hormone and the brain insulin-like peptides (ILP2, ILP3, ILP5). Other neuropeptides, such as insulin-growth factor ILP6, neuropeptide F, allatostatin-A, corazonin, leucokinin, tachykinins and limostatin, might stimulate lipolysis, while diapause hormone-pheromone biosynthesis activating neuropeptide, short neuropeptide F, CCHamide-2, and the cytokines Unpaired 1 and Unpaired 2 might induce lipogenesis. Most of these peptides interact with one another, but mostly with insulin signaling, and therefore affect lipid metabolism indirectly. Peptide hormones are also involved in lipid metabolism during reproduction, flight, diapause, starvation, infections and immunity; these are also highlighted. The review concludes with a discussion of the potential of lipid metabolism-related peptide hormones in pest management.

## Introduction

In all living being, carbohydrate, protein and lipids are the three main energy sources for vital activities of insects. Among these sources, lipids are the primary storage molecules and an essential source of energy for growth and development, reproduction, periods of prolonged flight, starvation, and diapause. Many insect sex pheromones, cuticular wax, as well as various defensive secretions, such as phenols, quinones and carboxylic acids, contain or are synthesized from lipids (Downer and Matthews, 1976; Klowden, 2007; Yew and Chung, 2015).

The center for insect lipid metabolism is the fat body, which is analogous to vertebrate adipose tissue and the liver. The fat body is primarily composed of adipocytes, which accumulate triacylglycerols (TAGs) in intracellular lipid droplets (LDs). Genomics and proteomics, together with functional analyses, such as RNA interference (RNAi) and CRISPR/Cas9-targeted genome editing, have revealed that storage of lipids and their metabolism are conserved, sophisticated and complicated processes. These studies identified various genes expressed by adipocytes that are involved in lipid metabolism and elucidated their functions. Briefly, lipid metabolism starts with the hydrolysis of the dietary lipids in midgut via lipases, lipid transport into target sites, primarily the fat body, and muscles and ovaries by lipophorins, cellular uptake and transport by fatty acid transport and fatty acid binding proteins, synthesis, accumulation and hydrolysis of lipids in the fat body by Fatty Acid Synthase (FAS) and perilipins. These processes occur at the mRNA level by transcription factors and post-transcriptional modifications of proteins. Most of these events are under the control of the insect endocrine system.

The insect endocrine system consists primarily of neurosecretory cells and endocrine glands (e.g., corpora cardiaca, corpora allata, and prothoracic glands). Organs such as the midgut, fat body, ovaries and testes are also considered endocrine glands as they synthesize various hormones. Insect hormones could be classified as amine-type (e.g., octopamine, serotonin, and tyramine), steroids (ecdysteroids), sesquiterpenes [juvenile hormone (JH)], peptide-type [e.g., prothoracicotropic hormone, adipokinetic hormone (AKH)] and lipid-type (e.g., prostaglandin). Among these, ecdysteroids and JHs are the most-studied and indirectly affect lipid metabolism due to their general effect on growth and development. However, the essential and key hormones affecting insect lipid metabolism are peptide-hormones.

Peptide hormones are central to many aspects of insect life, such as molting (Nässel et al., 2015), development (Oudejans et al., 1993), reproduction (Hou et al., 2017), digestion (Borovsky et al., 1990), behavior (Wu et al., 2003; Gospocic et al., 2017), and pheromone production (Sato et al., 1993), in addition to lipid metabolism. Peptide hormones are produced by neurosecretory cells and endocrine glands. Most of these hormones are produced by the central nervous system and specifically referred to as “neuropeptides.” Regardless of their origin, many peptide hormones perform their tasks by binding into their cognate G protein-coupled receptors (GPCRs) (Park and Adams, 2010; Duan-Şahbaz and Ýyison, 2018). Studies on the genome of the common fruit fly, *Drosophila melanogaster* (stated as *Drosophila* since here throughout the article), have revealed more than 35 neuropeptide and 45 GPCR genes (Duan-Şahbaz and Ýyison, 2018); similar numbers have been reported from other species (Wang et al., 2018).

Various peptide-hormones have been shown to affect insect lipid metabolism and the current review focuses on these peptide hormones and their role(s) in insect lipid metabolism. The role of these peptide hormones in lipid metabolism-related biological events, such as reproduction, flight, diapause, starvation, infection and parasitism including the potential of peptide hormones in pest management is discussed.

## Peptide Hormones Involved in Insect Lipid Metabolism

The major peptide hormones directly or indirectly involved in insect lipid metabolism are Adipokinetic Hormone (AKH), Insulin-like Peptides (ILPs), Diapause Hormone-Pheromone Biosynthesis Activating Neuropeptide (DH-PBAN), Short Neuropeptide F (sNPF), Neuropeptide F (NPF), Allatostatin-A (AstA), Corazonin (Crz), Leucokinin (Lk), CCHamide-2 (CCHa2), Tachykinins (Tk), Cytokines (Adipokines), and Limostatin (Lst) (Table 1).

**TABLE 1:** Peptide hormones involved in insect lipid metabolism and their features.

Name/Abbreviation	Major synthesis site	Other synthesis sites	Length (amino acid)	Roles	Lipid-specific role
Adipokinetic hormone (AKH)	Corpora cardiac	Ganglia located in ovaries, midgut, fat body, accessory glands and muscle	79- D. melanogaster	• Stimulation of heart beat • Increase of muscle tonus • Stimulation of general locomotion • Enhancement of immune response • Protection against oxidative stress • Mobilization of lipid stores for reproductive activities, flight, diapause preparation, and starvation	Lipolysis
**INSULIN-LIKE PEPTIDES**					
Brain insulin-like peptide 2 (ILP2)	Insulin producing cells in adult brain	Embryonic and larval midgut, salivary glands and mesoderm	137- D. melanogaster 136- L. decemlineata	• Regulation of carbohydrate metabolism • Inhibition of foxO activity • Activation of Sterol regulatory element-binding protein (SREBP) for *de novo* lipogenesis • Reproductive activities, fecundity • Lipid accumulation for diapause preparation	Lipogenesis
Brain insulin-like peptide 3 (ILP3)	Insulin producing cells in adult brain	Intestinal muscle	120- D. melanogaster	• Regulation of carbohydrate metabolism • Inhibition of foxO activity • Activation of Sterol regulatory element-binding protein (SREBP) for *de novo* lipogenesis • Lipid accumulation for diapause preparation	Lipogenesis
Brain insulin-like peptide 5 (ILP5)	Insulin producing cells in adult brain	Ovaries and Malpighian tubules	108- D. melanogaster	• Regulation of carbohydrate metabolism • Inhibition of foxO activity • Activation of Sterol regulatory element-binding protein (SREBP) for *de novo* lipogenesis • Lipid accumulation for diapause preparation	Lipogenesis
Insulin-like growth factor insulin-like peptide 6 (ILP6)	Larval and adult fat body	Salivary glands, heart and glial cells in the ventral nerve cord	107- D. melanogaster	• Suppression of brain ILPs • Induction of lipid uptake, activation of lipid turn over in oenocytes in fasting • Induction of starvation tolerance	Lipolysis
**Diapause hormone-pheromone biosynthesis activating neuropeptide (DH-PBAN)**	Neurosecretory cells in the subesophageal ganglion		192-B. mori 194-H. armigera (Active peptide: 24)	• Induction of diapause • Activation of extracellular signal-regulated kinase phosphorylation	Lipogenesis
**Short neuropeptide F (sNPF)**	Brain lateral neurosecretory cells	Midgut, hindgut, antennae, Malpighian tubules and ovary	281- D. melanogaster (Active peptide: 6–19)	• Regulation of feeding behavior • Locomotor activity Circadian rhythm • Appetitive olfactory behavior • Sleep homeostasis • Hormone release • Gut epithelial integrity • Stimulation of ovarian development	Lipogenesis
**Neuropeptide F (NPF)**	Brain	Subesophageal ganglion and midgut	102- D. melanogaster (Active peptide > 28)	• Regulation of feeding behavior and food choice • Adult longevity Wakefulness • Modulation of odor-aroused appetitive behavior • Reproduction • Suppression of the inhibitory influence of AstA activity	Lipolysis
**Allatostatin-A (AstA)**	Brain	Gut	151- D. melanogaster	• Inhibition of starvation-induced feeding behavior • Regulation of AKH and ILP signaling	Lipolysis
**Corazonin (Crz)**	Brain lateral neurosecretory cells		154- D. melanogaster (Active peptide: 11)	• Cardioactivity • Regulation of the ecdysis initiation • Melanization Stress responses Sperm transfer and copulation • Regulation of ethanol sedation • Induction of food uptake	Lipolysis (starvation-induced)
**Leucokinin (Lk)**	Brain, insulin producing cells and ventral ganglia		160- D. melanogaster (Active peptide: 6–15)	• Myotropic activity • Regulation of water and ion homeostasis in Malpighian tubules and hindgut • Meal size regulation • Regulation of feeding, metabolic rate, post-feeding physiology and behavior • Regulation of locomotor activity • Regulation of starvation-induced sleep suppression	Lipolysis (starvation-induced)
**CCHamide-2 (CCHa2)**	Fat body and midgut		136- D. melanogaster	• Stimulation of feeding • Stimulation of insulin signaling	Lipogenesis
**Tachykinin (Tk)**	Gut	Central nervous system	297- D. melanogaster	• Locomotor activity • Olfactory responses • Midgut immunity • Reduction of insulin signaling and lipid storage	Lipolysis
**Cytokines**					
Unpaired 1 (UPD1)	Brain		413- D. melanogaster	• Sensing of the fed-state • Activation of insulin signaling	
Unpaired (UPD2)	Fat body		406- D. melanogaster	• Sensing of the fed-state • Activation of insulin signaling	Lipogenesis in adipose tissue, Lipolysis in oenocytes
**Limostatin**	AKH-producing neurons in corpora cardiaca	Fat body	139- D. melanogaster	∙ Suppression of insulin production	Lipolysis

### Adipokinetic Hormone (AKH)

Adipokinetic Hormones (AKHs) are glucagon-like peptides and are produced by the neurosecretory cells of the corpora cardiaca (Beenakkers et al., 1985; Goldsworthy and Mordue, 1989; Goldsworthy et al., 1997; Table 1). These cells might be present both in larval and adult stages, and release AKH in response to developmental stage or conditions (Lee and Park, 2004). AKH is synthesized as a preprohormone consists of a hydrophobic signal peptide, a bioactive neuropeptide and an AKH-associated peptide, which is also known as AKH Precursor-Related Peptide (APRP) (Van der Horst et al., 2001; Van der Horst, 2003). The signal peptide is removed co-translationally and the remaining prohormone is stored in the CC. The bioactive peptide is cleaved from the prohormone prior to its release into the hemolymph (O’Shea and Rayne, 1992; Oudejans et al., 1999). The APRPs can be further processed to form smaller peptides; however, their exact role is not known (Baggerman et al., 2002; Huybrechts et al., 2002). AKH bioactive peptides have 8–10 amino acids, an amino terminus blocked by pyroglutamate, a carboxy terminus blocked by amidation, aromatic residues at positions 4 (Phe or Tyr) and 8 (Trp), and a Gly residue at position 9 (Gäde et al., 1997; Gäde, 2004; Gäde and Marco, 2013; Table 1). It is noteworthy that expression of AKH genes is not restricted to the corpora cardiaca as different mRNA variants are produced by the ganglia located in the ovaries, midgut, fat body, accessory glands and muscle tissues (Abdel-latief and Hoffmann, 2007; Kaufmann et al., 2009).

The first report on the presence of an AKH goes back 50 years describing the involvement of this peptide in the mobilization of lipids during flight in the migratory locust *Schistocerca gregaria* (Beenakkers, 1969; Mayer and Candy, 1969). However, the AKH signaling system has been studied mainly in the tobacco hornworm, *Manduca sexta*, and its AKH was first sequenced in 1985 (Ziegler et al., 1985). Not surprisingly, *Drosophila* is another model that adds significantly into our knowledge on AKHs (Grönke et al., 2007; Baumbach et al., 2014b; Gáliková et al., 2015). The AKH signaling system is also present in coleopterans, hemipterans, orthopterans, blattodeans and hymenopterans, and more than 60 different kinds of AKHs have been identified (Kaufmann and Brown, 2006; Konuma et al., 2012; Gäde and Marco, 2013; Marchal et al., 2018). The number and sequences of insect AKHs are diverse, for example, three different AKHs (AKH-I to III) with different bioactivities are present in the migratory locust, *Locusta migratoria* (Oudejans et al., 1993; Vroemen et al., 1998), whereas two AKHs are present in *S. gregaria* (Oudejans et al., 1991) and a single AKH is present in *Drosophila* (Grönke et al., 2007).

AKH has been shown to be involved in various events, such as the stimulation of heart beat (Scarborough et al., 1984), general locomotion (Socha et al., 1999), neuronal signaling (Milde et al., 1995), increase of muscle tonus (O’Shea et al., 1984), immunity (Goldsworthy et al., 2002), and protection of insects against oxidative stress (Bednářová et al., 2013). However, its primary role is to initiate the lipid/carbohydrate mobilization from the fat body (Van der Horst et al., 2001). The lipid mobilization occurs through the action of AKH on the Triglyceride Lipase (TGL) (Arrese et al., 2006; Arrese and Soulages, 2010). Additionally, there is a second system initiating lipolysis, the Brummer (bmm) lipase (homolog of mammalian Adipose Triglyceride Lipase, ATGL) (Grönke et al., 2005). *Bmm*-mutant flies are lipid mobilization-impaired and obese (Grönke et al., 2005). Notably, the AKH system functions in response to rapid changes in lipid demands, while bmm functions to maintain the lipid levels for a metabolic baseline (Grönke et al., 2007), therefore, bmm is also necessary during the periods of energy demand.

**FIGURE 1:**
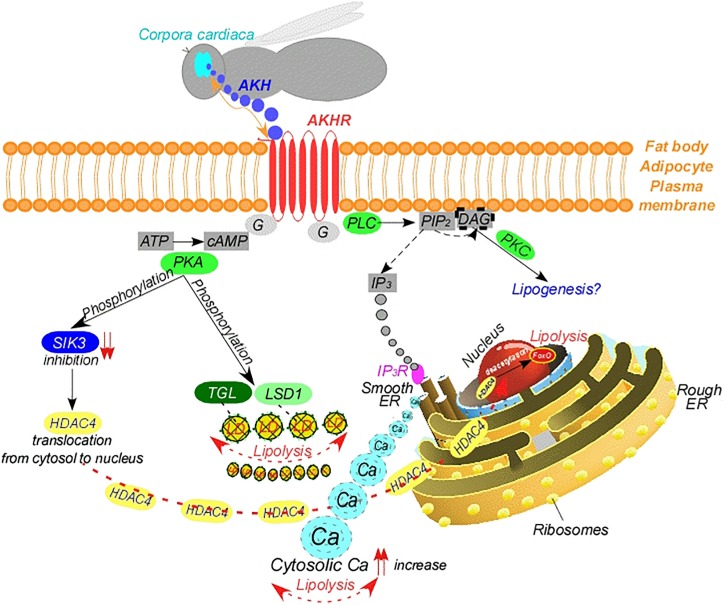
Mode of action of adipokinetic hormone. AKH, adipokinetic hormone; AKHR, adipokinetic hormone receptor; Ca, calcium; DAG, diacylglycerol; ER, endoplasmic reticulum; G, g-protein couple; HDAC4, histone deacetylase 4; IP_3_, inositol-1,4,5-trisphosphate; IP_3_R, inositol-1,4,5-trisphosphate receptor; LD, lipid droplet; LSD1, lipid storage droplet 1 protein; PLC, phospholipase C; PIP_2_, phosphatidylinositol 4,5-diphosphate; PKA, cAMP-dependent protein kinase; PKC, calcium-dependent protein kinase C; SIK3, salt induced kinase 3; TGL, triglyceride lipase.

AKH exerts its effects on lipid mobilization via signal transduction (Canavoso and Wells, 2001; Figure 1). AKH binds to its GPCR, the Adipokinetic Hormone Receptor (AKHR) related to the mammalian gonadotropin-releasing hormone receptor (Lindemans et al., 2009) and was first identified from *Drosophila* and the silkworm *Bombyx mori* (Park et al., 2002; Staubli et al., 2002). AKHR is produced primarily in the fat body (Arrese and Soulages, 2010), but also in other tissues, such as the midgut, muscles, brain and reproductive organs (Kaufmann and Brown, 2006; Ziegler et al., 2011; Zandawala et al., 2015; Alves-Bezerra et al., 2016; Hou et al., 2017). Binding of AKH to its cognate AKHR results in the activation of two different second-messenger systems involved in lipid mobilization (Park et al., 2002; Staubli et al., 2002; Figure 1). In one pathway, binding of AKH to AKHR leads to stimulation of Phospholipase C (PLC), which cleaves membrane lipid phosphatidylinositol 4,5-diphosphate (PIP_2_) into inositol-1,4,5-trisphosphate (IP_3_) and diacylglycerol (DAG). Finally, release of IP_3_ activates IP_3_ Receptor (IP_3_R) in the endoplasmic reticulum, leading to mobilization of the second messenger calcium from the endoplasmic reticulum to cytosol (Gäde and Auerswald, 2003; Figure 1). The increase in cytosolic concentrations of calcium transmits the AKH signal, however, the exact mechanism is not known (Arrese et al., 1999; Van der Horst et al., 1999; Baumbach et al., 2014a, b). In brief, calcium stored within the endoplasmic reticulum represents an important signal for lipid mobilization in the first pathway. In the second pathway, binding of AKH to its receptor on the fat body cell surface activates adenylate cyclase and mediates a rapid increase of the second messenger cyclic Adenosine monophosphate (cAMP) leading to activation of cAMP-dependent Protein Kinase (PKA), which promotes the phosphorylation of downstream elements, such as the LDs, TGL and Perilipin 1/Lipid Storage Droplet-1 Protein (PLIN1/LSD1) (Arrese and Wells, 1994; Arrese et al., 1999; Arrese et al., 2008; Figure 1). PLIN1 phosphorylation has been shown to increase the accessibility of LDs for TGL, thereby allowing lipid mobilization. In accordance with this, *Drosophila PLIN1*-mutants are obese (Beller et al., 2010). On the other hand, PKA inhibits the activity of a member of the AMP-activated Protein Kinase (AMPK) family, the Salt-Inducible Kinase 3 (SIK3) by phosphorylating a conserved serine residue (Ser^563^ in *Drosophila* SIK3) (Wang et al., 2011; Figure 1). This leads to translocation of a class IIa histone deacetylase, the Histone Deacetylase 4 (HDAC4), from cytosol into the nucleus, where it deacetylates and activates the transcription factor Forkhead Box Class O (foxO) (Wang et al., 2011; Choi et al., 2015; Figure 1). This results in the activation of foxO targets, such as bmm, leading to lipolysis. Overexpression of *HDAC4* leads to up-regulation of the *bmm*, indicating that HDAC4 regulates *bmm* expression in the fat body (Choi et al., 2015). In line with this, *SIK3*-null mutants exhibit a lipodystrophic (lean) phenotype and display up-regulated *bmm* expression and increased lipase activity as expected. By contrast, constitutive over-expression of active *SIK3* completely blocks the *bmm* expression (Choi et al., 2015). Interestingly, deletion of *SIK3* reversed both the lipid accumulation and the reduced *bmm* expression phenotypes of *AKHR*-mutant flies (Choi et al., 2015). It is noteworthy that a serine/threonine kinase known as Liver Kinase B1 (LKB1) also plays an important role in governing lipid metabolism by activating SIK3 in a kinase activity-dependent manner (Choi et al., 2015). Thus, *Drosophila LKB1*-mutants display decreased lipid storage and increased expression of *bmm*, suggesting that the AKH pathway inhibits the kinase activity of LKB1 (Choi et al., 2015). In line with this, foxO is dephosphorylated, therefore activated, and localized to the nucleus during lipolysis. In brief, the LKB1-SIK3 pathway is upstream of HDAC4, whereas LKB1, SIK3, HDAC4, and foxO are downstream elements of AKH signaling. Additionally, AKH signaling works in a manner opposite to LKB1-SIK3 signaling.

Null mutations in *AKH* or *AKHR* result in obese *Drosophila* adults, whereas their over-expression leads to a dramatic reduction of lipid stores (Grönke et al., 2007; Bharucha et al., 2008; Baumbach et al., 2014b; Gáliková et al., 2015). In accordance with this, LDs accumulated in adipocytes of *AKHR* deletion mutants (obese) and in flies subjected to RNAi-mediated knockdown of *AKHR* in the fat body. In contrast, LDs are largely depleted from adipocytes of the flies subjected to *AKH* or *AKHR* overexpression (lean). In accordance with this, silencing of *AKHR* reduces DAG levels leading to TAG accumulation in the fat body in the kissing bug *Rhodnius prolixus* (Alves-Bezerra et al., 2016), the two-spotted cricket *Gryllus bimaculatus* (Konuma et al., 2012) and the oriental fruit fly *Bactrocera dorsalis* (Hou et al., 2017). Additionally, double-mutant flies, which lack the lipolytic PLIN1 and AKHR, were found to be more obese than the single *PLIN1-* or *AKHR*-mutants and remained lipolysis-competent (Grönke et al., 2007; Beller et al., 2010). Dual knockout of the *AKHR* and *bmm* genes in *Drosophila* yields flies also that are obese and not starvation-tolerant (Grönke et al., 2007). Notably, overexpression of *AKH* in *bmm*-*Drosophila* mutants was still found to reduce the excessive TAG storage; however, *bmm* expression was found to be higher in *AKHR*-mutants, suggesting AKH/AKHR signaling is not a prerequisite for bmm activity (Grönke et al., 2007). Females of tsetse fly of which *bmm* or AKH/AKHR systems were silenced individually or together were found to have prolonged lifespan under starvation and elevated lipid levels at the time of death, suggesting extended survival is likely due to the reduced rate of lipolysis during starvation and the inability to utilize lipid reserves (Attardo et al., 2012). On the other hand, *bmm* expression shows an antagonistic response to disturbed AKH and fat body calcium homeostasis, as opposed to the lipogenic gene *midway* (*mdy*) encoding the Diacylglycerol O-Acyltransferase 1 (DGAT1) (Buszczak et al., 2002), which is down-regulated in response to increased cytosolic calcium levels in fat body cells (Bi et al., 2014). Conversely, depletion of cytosolic calcium levels in the fat body of adult flies up-regulates *mdy*, and down-regulates *bmm* expression (Baumbach et al., 2014a), suggesting AKH signaling via calcium promotes *bmm* expression (Baumbach et al., 2014a; Choi et al., 2015). In addition, AKH has been shown to be involved in the lipid mobilization only in the adult stage, but not in larval stages in *Drosophila* (Gáliková et al., 2015). Lee and Park (2004) also reported that fat body TAG content did not change in *AKH*-deficient mutants, suggesting that lipid metabolism might occur normally also in the absence of AKH.

The extracellular or intracellular calcium is important in the lipolytic response by AKH as mentioned above and has been demonstrated in the adults of the orthopterans *S. gregaria* (Ogoyi et al., 1998), *L. migratoria* (Auerswald and Gäde, 2006), *G. bimaculatus* (Anand and Lorenz, 2008), lepidopteran *M. sexta* (Arrese et al., 1999), the coleopterans *Pachnoda sinuata* (Auerswald et al., 2005) and *Zophobas atratus* (Slocinska et al., 2013) and *Drosophila* (Grönke et al., 2007; Baumbach et al., 2014a; Gáliková et al., 2015). For example, incubation of fat body from *P. sinuata* in calcium-free medium reduces the elevation of cAMP in comparison to that in calcium-containing medium, further indicating the importance of calcium in the AKH-induced lipolytic response (Auerswald and Gäde, 2001). However, no effect of calcium signaling on TAG levels has been detected in *Drosophila* larvae, which is in accordance with the fact that AKH mobilizes lipids only in the adult stage (Baumbach et al., 2014a; Gáliková et al., 2015).

Calcium homeostasis is primarily coordinated by a process called “Store-Operated Calcium Entry (SOCE),” which has two major components; the SERCA (Sarco/Endoplasmic Reticulum Calcium-ATPase) that pumps calcium from the cytosol into the endoplasmic reticulum lumen and the IP_3_R that releases calcium from endoplasmic reticulum into cytosol. Binding of AKH to AKHR triggers an IP_3_ second messenger response via GPCR signal transducer G-proteins, such as G Protein α q Subunit (Gαq) and G Protein γ 1 Subunit (Gγ1), and phospholipase C (PLC) (Baumbach et al., 2014b). Binding of IP_3_ to IP_3_R in the endoplasmic reticulum membrane causes calcium efflux, which is sensed by the “Stromal Interaction Molecule (STIM).” STIM interacts with the Plasma Membrane Calcium Channel Protein 1, Orai1, to elevate cytosolic calcium levels (Cahalan, 2009). At resting stage, STIM is bound to calcium and spread evenly throughout the endoplasmic reticulum membrane. Upon activation, STIM translocates to junctions between endoplasmic reticulum and plasma membrane, where it couples with Orai1. This coupling results in the import of calcium from the extracellular compartment to the cytosol, providing spatial calcium replenishment into the endoplasmic reticulum lumen through SERCA.

It is not surprising that genes involved in calcium homeostasis affect lipid metabolism when the calcium/AKH interaction is taken into consideration. For example, impaired SERCA activity leads to reduced fat storage in adipose tissue in *Drosophila* (Baumbach et al., 2014a). This appears to be opposite to the effects of impaired endoplasmic reticulum calcium homeostasis on fat storage in mammalian hepatocytes (Bi et al., 2014). On the other hand, loss of *IP_3_R* leads to obesity in *Drosophila* adults (Subramanian et al., 2013a, b). Chronic silencing of *STIM* leads to obesity and dysfunction of lipid mobilization due to reduced AKH signaling in adult fly fat body, whereas *AKHR* is up-regulated in the fat body of flies continuously expressing *STIM*, suggesting an impairment of AKH upon *STIM* disruption (Xu et al., 2019). In line with this, *bmm* was down-regulated and *mdy* was up-regulated upon *STIM* knock down, however, the *mdy* up-regulation was found only at day 1 of silencing (Xu et al., 2019). Functional impairment of the PLC, and Gαq, Gγ1, STIM, and AKHR, lowers the intracellular calcium concentration and increases the fly body TAG content (Baumbach et al., 2014b). At the onset of PLC-dependent adiposity, *mdy* was found to be up-regulated and *bmm* down-regulated (Baumbach et al., 2014b). Similarly, a Gγ1-dependent body fat increase correlated with an almost doubled expression of the lipogenic *mdy* gene and reduction of *bmm* expression. Over-expression of *G*α*q* or *STIM* leads to lean flies which down-regulated *mdy*, and up-regulated *bmm* (Baumbach et al., 2014b). Notably, silencing calmodulin (*CaM*) encoding a calcium-binding messenger protein in the adult fat body also leads to an increase in fly body TAG content as CaM is also regulated by intracellular calcium (Baumbach et al., 2014b). In addition, silencing *CaM* leads to a similar transcriptional response of the *mdy* (upregulation) and *bmm* (downregulation) genes (Baumbach et al., 2014b). In brief, changes in calcium homeostasis directly impact fat deposition and AKH signaling employs the Gαq/Gγ1/PLC/STIM module of GPCR-dependent calcium signaling to regulate lipid mobilization (Baumbach et al., 2014b). *G*α*q*, *G*γ*1*, *PLC*, *STIM*, *IP_3_R*, and *CaM* act as anti-obesity genes, whereas *SERCA* acts as an obesity gene.

**FIGURE 2:**
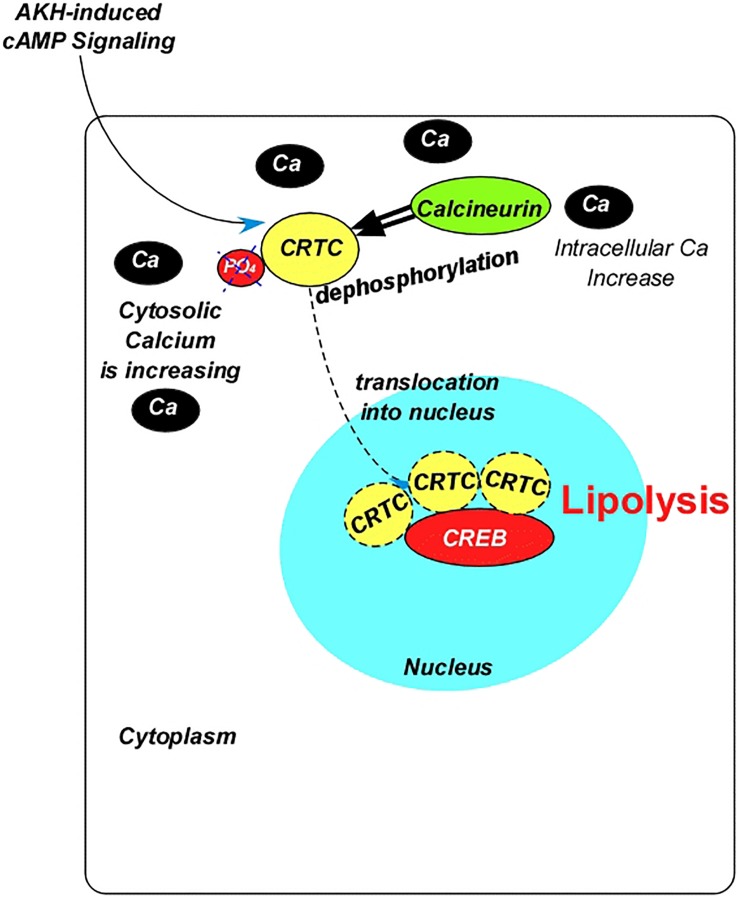
Diagram summarizing the interaction between the adipokinetic hormone and the transcription factor cAMP response element-binding protein. AKH, adipokinetic hormone; Ca, calcium; CREB, cAMP response element-binding protein; PO_4_, phosphate; CRTC, cAMP-regulated transcriptional co-activator.

GPCRs can also activate calcium-dependent Protein Kinase C (PKC) (Ojani et al., 2016) and Calcium/Calmodulin-dependent Protein Kinase II (CaMKII) (Van Marrewijk et al., 1991; Liu P. et al., 2015). The membrane-bound DAG produced by signal-induced activation of PLC could activate PKC, which might phosphorylate other molecules, leading to lipogenesis. However, the CaMKII is likely to lead to a lipolytic response. AKH signaling via CaMKII has been demonstrated to inhibit secretion of the adipokine Unpaired 2 (UPD2), which triggers systemic insulin signaling from the central brain (Rajan and Perrimon, 2012) and impairs TAG mobilization (Rajan and Perrimon, 2012; Rajan et al., 2017). Additionally, AKH induces the transcriptional factor cAMP Response Element-Binding Protein (CREB) via increased cAMP through PKA signaling (Iijima et al., 2009; Figure 2). This occurs through the activity of the CREB co-activator, “cAMP-Regulated Transcriptional Co-activator (CRTC),” which works in cooperation with the foxO during fasting in mammalians (Koo et al., 2005; Dentin et al., 2007; Matsumoto et al., 2007). The CRTC-related lipolytic response requires its dephosphorylation (at Ser^157^), which is accomplished by the calcium-dependent calcineurin (CaN), a calcium/calmodulin-dependent serine/threonine phosphatase that binds directly to CRTC (Wang et al., 2008; Yang et al., 2013; Figure 2). Thus, increases in intracellular calcium stimulate CREB target gene expression (Screaton et al., 2004; Koo et al., 2005) and induces CRTC dephosphorylation, therefore activation (Figure 2). As will be discussed under “Insulin-like Peptides,” ILPs inhibit CRTC activity in a phosphorylation-dependent manner, which occurs primarily in the feeding stages.

Genetic activation of AKH signaling suppresses the expression of lipogenic *midway* encoding the DGAT1. On the other hand, knockdown of *AKHR* leads to upregulation of the lipogenic gene encoding Acyl-CoA-Binding Protein-1 (ACBP1), which was shown in *R. prolixus*. ACBP1 is required for binding of the acyl-CoA produced from fatty acids that are released during TAG hydrolysis, as well as delivery to the acyltransferases involved in TAG synthesis (Alves-Bezerra and Gondim, 2012; Alves-Bezerra et al., 2016). Downregulation of the CREB target genes, *ACBP1* in this case, is in accordance with CRTC dephosphorylation. *AKHR* silencing leads to also downregulation of the gene encoding mitochondrial-like *GPAT1* (Glycerol-3-Phosphate-O-Acyltransferase 1) required for the first and committed step in the synthesis of TAG. This may be related to avoiding excessive TAG synthesis exceeding the cellular capacity of storage (Alves-Bezerra et al., 2010, 2016). *AKHR* knockdown leads to TAG accumulation in fat body and flight muscles, and reduced hemolymph lipid levels after starvation in *R. prolixus*, also indicating the requirement of AKHR in TAG mobilization (Zandawala et al., 2015; Alves-Bezerra et al., 2016). Notably, injection of the ligand, AKH, induces expression of both *ACBP1* and *GPAT1* when the AKHR is also highest in the fat body (Alves-Bezerra et al., 2016). Similarly, knockdown of *AKHR* in *B. dorsalis* resulted in TAG accumulation both in feeding and starving flies (Hou et al., 2017). In brief, cAMP and calcium signals stimulate CRTC dephosphorylation cooperatively through their effects on Salt-Inducible Kinases (SIKs) and phosphatases. Notably, other kinases, such as the Extracellular signal-Regulated Kinase 1 and 2 (ERK1 and ERK2) could be activated by GPCRs (Lim et al., 2015).

Obesity formed upon the impairment of AKH/AKHR has also been examined in terms of feeding behavior. Adipose tissue dysfunction promotes hyperphagia, which may be related to increased secretion of AKH (Xu et al., 2019). Thus, silencing *AKHR* causes hyperphagia in *G. bimaculatus*, while reducing hemolymph lipid levels (Konuma et al., 2012). In *Drosophila*, *AKHR* knockdown reduces fat body intracellular calcium leading to obesity as mentioned before. The obesity formed by functional impairment of STIM also depends on hyperphagia (Xu et al., 2019). The STIM-dependent hyperphagia is related to the remote up-regulation of the orexigenic *sNPF* gene that is expressed in the central nervous system (Baumbach et al., 2014a, b). Thus, over-expression of *sNPF* increases both food consumption and overall body size, whereas loss of *sNPF* decreases food intake (Lee et al., 2004). Notably, neurons use both extracellular and intracellular sources of calcium (Berridge et al., 2000; Bednářová et al., 2013). In this manner, various peptide hormones trigger IP_3_-induced release of calcium from non-mitochondrial intracellular storage compartments (Berridge and Irvine, 1989). On the other hand, there are controversial results in regard to the effect of AKH on feeding. Gáliková et al. (2017) reported a decrease in food intake in adult *Drosophila* upon *AKH/AKHR* mutation. Expression of *sNPF* has been reported to be unaffected by *AKH* mutation or by the *AKH* overexpression. However, another orexigenic peptide, Neuropeptide F (NPF), which encodes the fly counterpart of the mammalian orexigenic Neuropeptide Y (Nässel and Wegener, 2011), is up-regulated in *AKH*-mutants (Gáliková et al., 2017). Therefore, other mechanisms unrelated to AKH secretion could affect food uptake.

AKH interferes with the expression of other neuropeptide genes, such as Tachykinin (*Tk)*, Corazonin (*Crz)*, and Limostatin (*Lst)* (Gáliková et al., 2017). *Tk*, which encodes a hormone that positively regulates expression of *ILP2* and *ILP5* (Birse et al., 2011), is up-regulated in the fly gut upon food deprivation (Song et al., 2014). *AKH*-mutants have upregulated *Tk* mRNA levels. Nevertheless, overexpression of *AKH* is not sufficient to downregulate *Tk* (Gáliková et al., 2017). Tk is a negative regulator of fat storage (Song et al., 2014) and the increased expression of this gene in the *AKH*-mutants indicates that the de-repression of *Tk* might contribute to AKH deficiency-triggered obesity (Gáliková et al., 2017). On the other hand, genes encoding the cardioacceleratory peptides *Crz* and *Lst* are down-regulated in *AKH*-mutants, suggesting that other interactions likely to affect lipid metabolism (Xu et al., 2019). Thus, partial loss of *STIM* has been found to reduce Crz signaling leading to impaired larval development which might affect lipid metabolism (Megha Wegener and Hasan, 2019).

### Insulin-Like Peptides (ILPs)

Similar to mammalian insulin, insect Insulin-like Peptides (ILPs) are able to regulate circulating levels of carbohydrates in the hemolymph (Wu and Brown, 2006), thus, their temporal production is increased by hemolymph carbohydrate levels and decreased by starvation. ILPs are key elements of insect growth, reproduction, regulation of stress responses and life span. ILPs are primarily produced by the medial or lateral neurosecretory cells, known also as the insulin producing cells (IPCs) of the brain and the corpora cardiaca (Cao and Brown, 2001; Ikeya et al., 2002). Thus, the insulin signaling pathway in insects links metabolism and growth with the availability of nutrients. The fat body could also remotely control the secretion of ILPs from the IPCs through the Target of Rapamycin (TOR) pathway (Colombani et al., 2003; Géminard et al., 2009).

The classification of insect ILPs is based on similarities in the amino acid sequence of mature peptides to those of mammalian insulins, especially the number and locations of cysteine residues (Brogiolo et al., 2001; Grönke et al., 2010). Another conserved feature is the arrangement of the precursor (pre-proinsulin) protein with B-C-A domains that can be processed into dimeric peptides with an A and a B-chain linked by disulfide bridges (Nässel and Vanden Broeck, 2016). An exception to this structure has been detected for the insulin-like growth-factors (IGFs), where a short C-peptide is retained and the extended peptide is a single chain with internal cysteine bridges (Nässel and Vanden Broeck, 2016).

The first ILP to be identified in insects was bombyxin, or small prothoracicotropic hormone (Yoshida et al., 1998), and many ILPs from a variety of insects have been reported since. In *Drosophila*, eight ILPs [*Drosophila* Insulin-like Peptide 1-8 (DILP1-8)] (Kannan and Fridell, 2013), but only two receptors, a tyrosine kinase (Brogiolo et al., 2001) and the relaxin receptor-like leucine-rich repeats (Colombani et al., 2015) are found. DILP2, DILP3 and DILP5 resemble mammalian insulins and are primarily produced by IPCs in the adult brain and are therefore, denoted as “brain ILPs” (Brogiolo et al., 2001; Rulifson et al., 2002; Table 1). *DILP2* is also expressed in the embryonic and larval midgut, salivary glands and mesoderm (Brogiolo et al., 2001; Table 1). *DILP3* and *DILP5* transcripts are not detectable until larval stages (Brogiolo et al., 2001). *DILP3* is also expressed by the intestinal muscle (Veenstra et al., 2008), and *DILP5* is expressed in ovaries and Malpighian tubules (Ikeya et al., 2002; Table 1). DILP6 resembles IGFs structurally and functionally, and is produced in the larval and adult fat body, as well as in the salivary glands, heart and glial cells in the ventral nerve cord (Okamoto et al., 2009; Slaidina et al., 2009; Table 1). DILP7 and DILP8 have been proposed to be relaxin-like peptides (Yang et al., 2008; Colombani et al., 2012; Garelli et al., 2012). *DILP7* is expressed in the embryonic midgut during development and abdominal ganglia in third instar larvae and adults (Rulifson et al., 2002; Veenstra et al., 2008; Yang et al., 2008). *DILP8* is primarily expressed in the imaginal discs of the larva and ovaries of adults (Colombani et al., 2012; Garelli et al., 2012; Nässel et al., 2015) and shown to coordinate *Drosophila* tissue growth by delaying the onset of metamorphosis (Colombani et al., 2012; Garelli et al., 2012). *DILP1* is primarily expressed in IPCs mainly during the pupal stage, as well as in the adult stage (Slaidina et al., 2009; Liu W. et al., 2016). *DILP4* is expressed in the embryonic midgut and mesoderm during late-stage embryogenesis (Brogiolo et al., 2001). Together, this suggests that different ILPs are produced in different cell types and tissues at different developmental stages and may have multiple roles in other pathways (Nässel and Vanden Broeck, 2016).

**FIGURE 3:**
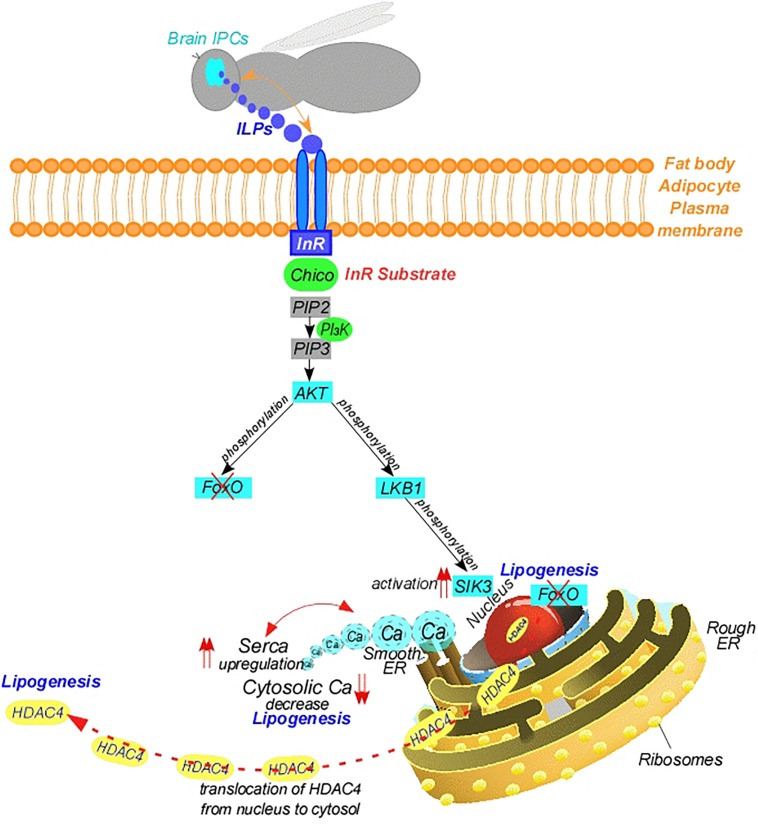
Mode of action of brain insulin-like peptides. AKT, serine-threonine protein kinase; Ca, calcium; ER, endoplasmic reticulum; G, g-protein couple; HDAC4, histone deacetylase 4; ILP, insulin-like peptide; InR, insulin-receptor; IPC, insulin producing cells; LKB1, liver kinase B1; PI_3_K, phosphoinositide-3-kinase; PIP_2_, phosphatidylinositol 4,5-diphosphate; PIP_3_, phosphatidylinositol 3,4,5-trisphosphate; SERCA, sarco/endoplasmic reticulum calcium-ATPase; SIK3, salt induced kinase 3.

The insulin signaling pathway appears to be highly conserved in insects (Figure 3). It is expected that the ILPs act similarly to insulin. When nutrients are abundant, the pathway is activated as ILPs released from the brain bind to an “Insulin Receptor (InR)” at the cell membrane (Fernandez et al., 1995; Chen et al., 1996). This leads to the recruitment of the InR substrate, Chico (Böhni et al., 1999), and subsequent activation of class I Phosphoinositide-3-Kinase (PI_3_K), which catalyzes the addition of a phosphate group to PIP_2_ forming phosphatidylinositol 3,4,5-trisphosphate (PIP_3_) (Oldham et al., 2002; Brown and Auger, 2011; Figure 3). The elevated PIP_3_ recruits Protein Kinase B (PKB), also known as Serine-Threonine Protein Kinase (AKT) to the membrane (Verdu et al., 1999; Britton et al., 2002). AKT can directly inhibit foxO activity by phosphorylation (Puig et al., 2003; Wang et al., 2008; Figure 3). An indirect route through AKT occurs through the activation of SIK3. In this route, AKT phosphorylates LKB1, and LKB1 phosphorylates and activates AMPKs, including SIK3 (Thr^196^ in *Drosophila* SIK3) (DiAngelo and Birnbaum, 2009; Funakoshi et al., 2011; Choi et al., 2015; Figure 3). In line with this, depletion of AKT enhances the activity of the CREB Co-activator, CRTC; therefore, insulin signaling pathway inhibits CRTC activity (Wang et al., 2008). Furthermore, over-expression of *LKB1* increases the level of phosphorylated AMPK (Funakoshi et al., 2011). This results in the phosphorylation and inhibition of HDAC4 by LKB1-activated SIK3 in the fat body, leading to dissociation of the HDAC4 from nucleus to the cytosol and inhibition of the lipolytic foxO. Thus, loss of *SIK3* leads to elevated expression of *bmm* and decreased lipid stores (Wang et al., 2011; Choi et al., 2015). Notably, *Drosophila* ILPs induce AKT-mediated SIK3 phosphorylation independently of increasing LKB1 kinase activity (Dentin et al., 2007; Wang et al., 2011; Choi et al., 2015). Thus, overexpression of *LKB1* induces lipid levels and downregulates *bmm*, suggesting LKB1 plays a critical role in lipid storage (Choi et al., 2015). On the other hand, AKT indirectly regulates TOR, a central regulator of cellular metabolism. In this manner, activation of AMPK leads to down-regulation of TOR signaling (Shaw et al., 2004) and phosphorylation of Raptor, a component of the TOR complex (Gwinn et al., 2008). Therefore, LKB1 suppresses TOR activity (Dentin et al., 2007). Briefly, in either route resulting in foxO inhibition, directly by AKT or indirectly via LKB1/SIK3, the inhibition of foxO leads to decrease in bmm activity, which leads to accumulation of lipids during feeding (Puig et al., 2003; Wang et al., 2011; Choi et al., 2015). This is in accordance with the increase in insulin in feeding stages. These findings suggest that foxO plays a central role in connecting insulin signaling to TAG metabolism (Heier and Kühnlein, 2018; Figure 3). By contrast, *bmm* is up-regulated when insulin signaling is low (Wang et al., 2011; Lee and Dong, 2017). Thus, reduction of insulin signaling, for example in starvation, stimulates dephosphorylation and nuclear translocation of foxO (Jünger et al., 2003; Puig et al., 2003), which in turn up-regulates genes encoding lipases involved in TAG hydrolysis (Vihervaara and Puig, 2008; Wang et al., 2011), supplying energy to the insect (Figure 1). In brief, the insulin-induced kinase activity of SIK3 controlled by LKB1 is critical for lipid storage in the fat body (Choi et al., 2015; Figure 3).

**FIGURE 4:**
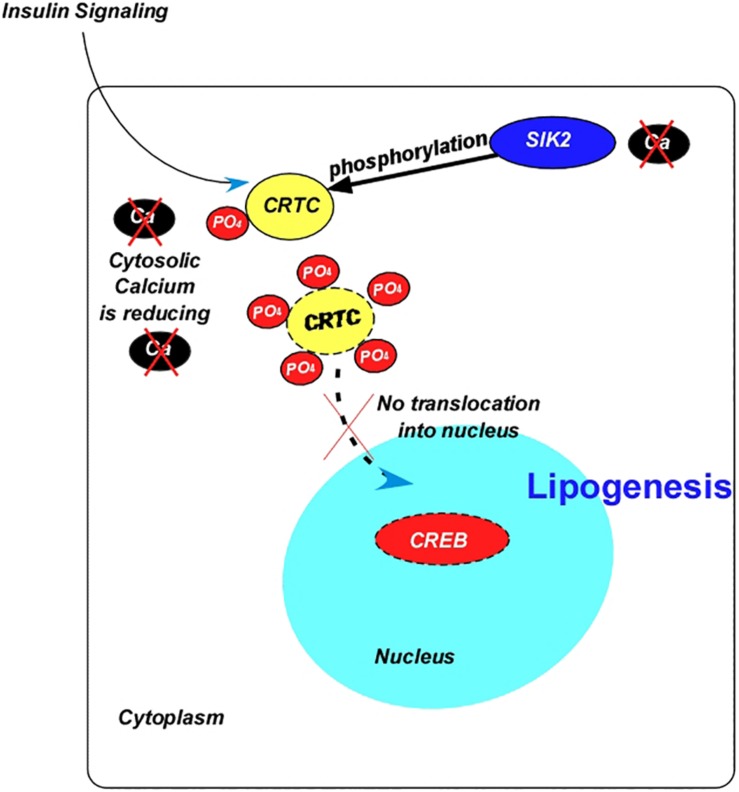
Diagram summarizing the interaction between the insulin signaling and the transcription factor cAMP response element-binding protein. Abbreviations: Ca, calcium; CREB, cAMP response element-binding protein; PO_4_, phosphate; SIK2, salt induced kinase 2; CRTC, cAMP-regulated transcriptional co-activator.

As mentioned before, the transcription factor, CREB induces lipolytic responses via the action of AKH. In accordance with this, down-regulation of *CREB* in the fat body leads to obesity in flies (Iijima et al., 2009). CREB also serves as a transcriptional factor target of ILPs, which occurs via the CREB co-activator CRTC (Figure 4). In parallel to the increase in insulin signaling, CRTC activity is inhibited during feeding through the phosphorylation of Ser^157^ by the Salt-Inducible Kinase 2 (SIK2) (Wang et al., 2008) leading to lipid accumulation. -Mutation of the inhibitory PKA phosphorylation site at Ser^1032^ to Ala in SIK2 further increased the amount of phosphorylated CRTC (Wang et al., 2008). Notably, CRTC is dephosphorylated by CaN, and SIK2 is inhibited during starvation (Figure 4). Additionally, deletion of *CRTC* induced the lethality of *LKB1-* and *SIK3-*null mutants (Choi et al., 2015).

Brain ILPs, and the ILP6 that suppresses the production of brain ILPs, are the most commonly studied ILPs (Table 1). Knock down of ILPs leads to various defects, such as loss of weight, reduced fecundity and body size, impaired development and metamorphosis or even lethality (Fernandez et al., 1995; Chen et al., 1996; Okamoto et al., 2009; Slaidina et al., 2009; Grönke et al., 2010; Fu et al., 2016; Deng et al., 2018), indicating their critical role in insect survival. Transgenic ablation of ILP-producing neurons in *Drosophila* leads to elevation of total blood sugar (Rulifson et al., 2002). Not surprisingly, ILPs are also key regulators of lipid metabolism (Broughton et al., 2005; DiAngelo and Birnbaum, 2009) and insulin signaling promotes TAG accumulation (DiAngelo and Birnbaum, 2009; Lehmann, 2018). This is indeed a complex interaction influenced by multiple factors, such as transcription factors, neuropeptides, neurotransmitters, lipases, and the internal interaction among ILPs and other actors originating from fat body and midgut. Basically, insulin (brain ILPs) inhibits the activity of foxO and activates the Sterol Regulatory Element-Binding Protein (SREBP), a transcription factor that regulates a variety of genes involved in *de novo* lipogenesis, leading to the accumulation of reserves. Not surprisingly, genes encoding several lipases and acyl-CoA synthetase or acyl-CoA dehydrogenases are also down-regulated by dietary sugars, therefore, repressed by the action of ILPs via foxO (Zinke et al., 2002; Wang et al., 2011; Mattila et al., 2015). Feeding on a high carbohydrate diet also induces the expression of genes encoding other lipogenic enzymes, such as Acetyl-CoA Carboxylase (ACC) and FAS (Zinke et al., 2002; Musselman et al., 2013; Mattila et al., 2015), which is coordinated by Mondo/Bigmax. This transcription factor likely binds to the promoter of *FAS* and/or *ACC* as *Bigmax*-mutant *Drosophila* larvae have decreased *ACC* and *FAS* gene expression, and store less TAG (Mattila et al., 2015). In addition, Mondo/Bigmax controls expression of genes encoding other transcription factors, such as *Sugarbabe*, which is highly induced by sugar and positively regulates lipogenic gene expression (Zinke et al., 2002; Mattila et al., 2015). Notably, reducing Mondo/Bigmax also reduces Seven-up, a positive regulator of insulin signaling (King-Jones and Thummel, 2005). Seven-up promotes glucose clearance and lipid turnover by inhibiting ecdysone signaling in the larval fat body (Musselman et al., 2018b). On the other hand, ablation of the IPCs, which leads to elimination of DILP2, DILP3, and DILP5 synthesis results in elevated levels of circulating carbohydrates levels in the hemolymph (hyperglycemia), elevated lipid storage in the fat body, increased starvation resistance, and extended life span in *Drosophila* adults (Brogiolo et al., 2001; Rulifson et al., 2002; Broughton et al., 2005). Likewise, silencing genes encoding ILPs increases the levels of lipid and carbohydrate in the hemolymph in *R. prolixus* (Defferrari et al., 2016) and *Spodoptera exigua* (Kim and Hong, 2015). In another study, elevated levels of TAG and obesity in *IP_3_R*-*Drosophila* mutants were also rescued after insulin expression, further suggesting the involvement of ILPs in lipid metabolism (Subramanian et al., 2013b). On the other hand, *DILP2*-knockdown leads to an up-regulation in *DILP3* and *DILP5* levels (Brogiolo et al., 2001), suggesting a compensatory balance mechanism amongst brain ILPs. Furthermore, the IGF-like DILP6 represses brain ILPs, thus, its over-production leads to a decrease in the expression of *DILP2* and *DILP5* in the brain, and DILP2 level in hemolymph in adult flies (Bai et al., 2012). In accordance with this, deletion of brain ILPs leads to up-regulation of *DILP6* in the fat body; however, *DILP6* deletion was not found to change the expression of brain DILP genes (Zhang and Xi, 2015). *DILP6* is also under the control of foxO and strongly induced upon starvation in a foxO-dependent manner in the larval fat body (Slaidina et al., 2009). During late larval and pupal stages when insects do not feed, *DILP6* expression is strongly induced (Okamoto et al., 2009; Slaidina et al., 2009). Thus, *DILP6*-mutants were shown to have elevated lipid levels (Grönke et al., 2010). As expected, loss of *DILP6* does not affect lipid stores in feeding larvae (Slaidina et al., 2009). Furthermore, DILP6 induces lipid uptake in oenocytes in fasting adult flies, indicating it is required for lipid turnover when adult flies are starved (Chatterjee et al., 2014). *DILP3* and *DILP5* expression is down-regulated, while the TAG levels increased in *miR-14*-mutant flies; however, the hyperlipidemic defect in *miR-14*-mutants was rescued by over-expressing *DILP3* (Varghese et al., 2010). This suggests an indirect role for DILP3 in lipid metabolism. In brief, high-sugar feeding promotes lipid biosynthesis and inhibits lipid catabolism to channel excess carbon derived from sugars into TAGs (Mattila and Hietakangas, 2017).

Insulin Receptor (InR) is essential to insulin activity, but also affects lipid storage as up-regulation of *Drosophila InR* specifically increases TAG stores in the adult fat body (DiAngelo and Birnbaum, 2009). Larvae over-expressing *InR* accumulate more fat in the fat body (Britton et al., 2002). Additionally, *InR* knockdown increases lipid levels in the hemolymph, while reducing lipid content in the fat body in *R. prolixus* (Defferrari et al., 2018). Interestingly, the levels of carbohydrates in the hemolymph and the fat body were found to be unchanged. The activation of AKT and phosphorylation of foxO were also reduced in knockdown insects (Defferrari et al., 2018). *InR* expression was found to be up-regulated in response to the reduction of ILPs in 5th instar *M. sexta* larvae (Walsh and Smith, 2011). In *L. decemlineata*, knockdown of *ILP2* led to up-regulation of *InR* and the insulin signaling target *Thor* gene encoding the translational regulator 4E Binding Protein (4EBP) (Fu et al., 2016). *4EBP* was also induced in *Drosophila ILP6*-mutants (Grönke et al., 2010). Furthermore, *4EBP* was significantly up-regulated in these mutants when combined with knockout of brain ILPs (Grönke et al., 2010). On the other hand, the InR substrate chico, an important component of the insulin signaling pathway, also affects lipid storage. For example, *Drosophila chico*-mutant males had an almost twofold increase in lipid levels despite their size reduction compared to normal flies (Böhni et al., 1999). The Chico binding protein SH2B, a SH2 domain-containing adaptor protein directly promotes insulin signaling, therefore, its disruption decreases insulin signaling and increases hemolymph carbohydrate levels, whole-body lipid levels, suggesting SH2B in fat body plays a key role in regulating lipid metabolism and energy homeostasis (Böhni et al., 1999; Song et al., 2010). In brief, InR is involved in fat body lipid storage in both non-feeding stages and post-feeding stages. These effects are likely to be regulated by the activation of AKT in a manner similar to mammalian insulin signaling pathway.

The cell growth factor, myc, in the fat body was also shown to facilitate DILP2 release from the brain, induce accumulation of TAGs and confer resistance to starvation (Gallant, 2013; Parisi et al., 2013). DILP2 accumulates in the IPCs of *Drosophila* larvae after starvation; however, reduced myc levels in the fat body also lead to accumulation of DILP2 in the IPCs during feeding, whereas increased myc levels decreases the amount of trehalose in the hemolymph (Parisi et al., 2013). Furthermore, down-regulation of genes encoding brain ILPs blocks the effect of myc on systemic growth, suggesting that myc activity in the fat body requires ILPs to induce systemic growth. As another point, expression of the gene encoding the fat desaturase Desat1, an enzyme that is necessary for monosaturation and production of fatty acids, was found to be necessary for myc-induced TAG storage (Parisi et al., 2013).

AKH signaling can also affect insulin signaling and, therefore, affect lipid metabolism (Buch et al., 2008; Hentze et al., 2015; Gáliková et al., 2017). AKH activity is antagonistic to insulin activity (Rulifson et al., 2002), thus, AKH elevates hemolymph trehalose titers (Park and Keeley, 1995). Additionally, *AKH*-mutants have increased expression of genes encoding brain ILPs, whereas *AKH* over-expression decreases their transcription. AKH signaling has been also reported to be required in the IPCs for sugar-dependent ILP3 release in *Drosophila* (Kim and Neufeld, 2015). However in another study, overexpression of *AKH* did not alter trehalose levels in adult *Drosophila* (Lee and Park, 2004). Nevertheless, silencing of *AKH* or ablation of the corpora cardiaca producing AKH inhibits fat body TOR activation in response to trehalose (Buch et al., 2008; Kim and Neufeld, 2015). This indicates that trehalose stimulates the corpora cardiaca to release AKH and AKH then acts directly on the IPCs to induce secretion of ILP3 leading to stimulation of TOR signaling (Kim and Neufeld, 2015). Therefore, the TOR pathway is directly activated by insulin. In line with this, *TOR*-mutant flies possess reduced fat body TAG levels, with a concomitant down-regulation in the lipogenic *Drosophila FAS* and an up-regulation in the lipolytic *bmm* (Luong et al., 2006). Furthermore, these mutants have also decreased hemolymph sugar levels, suggesting a sugar-regulatory role for TOR in addition to its involvement in the control of lipid metabolism (Luong et al., 2006). In accordance with these findings, adult flies lacking AKH are more resistant to starvation and do not exhibit starvation-induced hyperactivity (Lee and Park, 2004). Not surprisingly, ILP6 was found to be affected by the AKH deficiency in the opposite manner, thus, *AKH*-mutants have decreased *ILP6* mRNA (Gáliková et al., 2017) and, therefore, the obese phenotype in *AKH*-mutants could be also related to ILP6 reduction (Grönke et al., 2010; Gáliková et al., 2017). Briefly, ILP release by IPCs is stimulated by trehalose-activated AKH signaling leading to TOR activation (Kim and Neufeld, 2015).

One other factor that affects insulin signaling is the Insulin-related Peptide Binding Protein (IPBP), a homolog of the mammalian Insulin Growth-Factor Binding Protein (IGFBP) (Honegger et al., 2008). An IPBP, the Imaginal Morphogenesis Protein-Late 2 (ImpL2), which is a neural/ectodermal development factor in *Drosophila*, has been identified from cell culture of imaginal discs (Zapf et al., 1985; Honegger et al., 2008). In *Drosophila*, ImpL2 has been shown to bind to ILP2 and ILP5 and acts as a secreted antagonist of insulin signaling, as well as being essential for tolerance to starvation stress (Honegger et al., 2008). However, ImpL2 promotes insulin signaling in a subset of neurons in the larval brain (Bader et al., 2013). Sloth Andersen et al. (2000) identified a lepidopteran IPBP, which was also shown to be capable of inhibiting human insulin action at its receptor. Interestingly, ImpL2 is up-regulated in obese *AKH*-*Drosophila* mutants, suggesting that the peripheral insulin signaling decreases in response to AKH deficiency (Gáliková et al., 2017). As the expression pattern of the peripheral insulin targets does not reflect the increased expression of brain ILPs, up-regulation of ILPs could be a compensatory mechanism reflecting insulin resistance of *AKH*-mutants (Gáliková et al., 2017). Thus, AKH may act as an ILP antagonistic hormone by releasing foxO leading to the activation of genes involved in lipolysis and fatty acid oxidation.

Insulin signaling pathway also interferes with ecdysone and JH signaling (Nässel et al., 2015). FoxO plays a key role in these interactions, for example, higher concentrations of 20-hydroxyecdysone (20E) repress insulin-induced gene expression in the cotton bollworm, *Helicoverpa armigera* (Liu C.Y. et al., 2015). 20E antagonizes insulin signaling by up-regulating Phosphatidylinositol-3,4,5-Trisphosphate 3-Phosphatase (PTEN) expression, which represses AKT phosphorylation, thereby repressing foxO phosphorylation, leading to foxO nuclear localization and lipolysis (Rusten et al., 2004; Colombani et al., 2005; Figure 3). On the other hand, ILPs stimulates growth of prothoracic gland and/or ecdysone biosynthesis and release in *Drosophila* (Colombani et al., 2005), *B. mori* (Gu et al., 2009), *R. prolixus* (Vafopoulou and Steel, 1997), and *M. sexta* (Kemirembe et al., 2012). Moreover, ILPs also activate ecdysteroidgenesis in ovaries in the yellow fever mosquito, *Aedes aegypti* and *Drosophila* (Brown et al., 2008; Wen et al., 2010). Similarly, knocking down ILP2 was found to decrease 20E titer and repressed the expression of two 20E-response genes, those encoding the nuclear receptors HR3 (Hormone Receptor 3) and FTZ-F1 (Fushi Tarazu Factor 1) in *L. decemlineata* (Fu et al., 2016). Conversely, insulin signaling inhibits foxO activity by phosphorylation and 20E by controlling the expression of the gene encoding the transcriptional co-activator, “Diabetes and Obesity Regulated (DOR)” during feeding (Francis et al., 2010). Additionally, the relaxin-like ILP8, which is produced and secreted from abnormally growing imaginal discs, has been shown to delay metamorphosis by suppressing ecdysone biosynthesis in developing larvae (Garelli et al., 2012). On the other hand, the link between JH and insulin signaling was first demonstrated in *Drosophila* as *InR*-mutants were found to possess reduced JH biosynthesis (Tatar et al., 2001). Similarly, knockdown of *ILP2* in *L. decemlineata* resulted in a decrease in JH titers, as well as impaired pupation and adult emergence (Fu et al., 2016). In accordance with this, the levels of an allatostatin (Ast-C), which inhibits JH synthesis, were significantly increased upon silencing of *ILP2* in *L. decemlineata* (Meng et al., 2015; Fu et al., 2016). Knockdown of *ILP2* in the 3rd instar larvae also significantly reduced the transcript levels of the early JH target gene Krüppel-homolog 1, a zinc finger transcription factor, and a JH biosynthesis gene encoding the Juvenile Hormone Acid Methyltransferase (JHAMT) (Fu et al., 2016). Thus, knockdown of *ILP2* delayed the onset of the wandering in *L. decemlineata* larvae (Meng et al., 2019). Additionally, genes encoding the InR substrate *chico* and *PI_3_K*, which meditate insulin signaling, were also down-regulated upon *ILP2* silencing (Deng et al., 2018; Figure 3). In line with this, knockdown of *Chico* or *PI_3_K* reduced expression of several 20E- [*EcR* (Ecdysone Receptor), *HR3* and *E75* (Ecdysone-induced Protein 75)] and JH- [JHAMT, *Kr-h1* (Kruppel Homolog 1) and *Hairy*] signaling genes, leading to retardation of larval development and inhibition of larval growth (Deng et al., 2018). In another study, insulin was found to stimulate JH production in the German cockroach, *Blattella germanica* (Suren-Castillo et al., 2012). Notably, insulin signaling might modulate JH synthesis by affecting the allatotropins that stimulate JH production (Klowden, 2007). Therefore, JH and insulin signaling appear to interact through a positive feedback loop (Fu et al., 2016). It is noteworthy that foxO is also a critical factor in the regulation of lipid metabolism by JH, which was shown in tsetse flies (Baumann et al., 2013) and diapausing mosquitoes (Sim and Denlinger, 2013). These data all together suggest that the brain ILPs triggers JH signaling pathway during larval feeding and activates 20E signaling pathway at the late stage onset molting. The interaction between insulin, ecdysone and JH pathways affect the regulation of lipid metabolism.

Phosphatidic Acid Phosphatase (PAP), also known as lipin, converts phosphatidic acid into DAG, and therefore is also an important factor for insulin signaling (Finck et al., 2006; Schmitt et al., 2015). Thus, insulin signaling positively affects the role of lipins in LD formation (Schmitt et al., 2015). In *Drosophila*, lipin is localized to cytosol or nucleus (Valente et al., 2010), however, it translocates into the cell nucleus when nutrient availability and TOR signaling are low (Schmitt et al., 2015). Notably, down-regulation of the insulin pathway does not lead to nuclear translocation of lipin (Schmitt et al., 2015). Instead, reduced InR activity strongly promotes the small LD phenotype observed after reduction of lipin (Lehmann, 2018). In addition, reduced expression of *lipin* or knockdown of the *GPAT4* (Glycerol-3-Phosphate-O-Acyltransferase 4) and *AGPAT3* (1-Acylglycerol-3-Phosphate Acyltransferase 3, also known as Lysophosphatidic Acid O-Acyltransferase 3), the genes encoding enzymes preceding the dephosphorylation of phosphatidic acid by lipin, decreases PIP_3_ levels in the fat body. In accordance with this, impaired signaling through the InR-controlled PI_3_K-AKT pathway leads to increased hemolymph sugar levels in *Drosophila* larvae (Schmitt et al., 2015). In another study, a *GPAT4*-mutant was found to exhibit elevated levels of *DILP2* and *DILP3* mRNA, and decreased insulin responsiveness (Yan et al., 2015). In brief, PAP activity and an intact glycerol-3 phosphate pathway are required for regular insulin signaling (Schmitt et al., 2015).

As mentioned before, the gene encoding the leptin-like cytokine, UPD2, is induced in the adult fat body in response to either a high-sugar or a high-fat diet, and promotes systemic insulin secretion from IPCs (Rajan and Perrimon, 2012; Zhang and Xi, 2015). This occurs through the activation of the JAK/STAT (Janus Kinase/Signal Transducers and Activators of Transcription) signaling cascade in GABAergic neurons (Géminard et al., 2009; Rajan and Perrimon, 2012). Thus, knockdown of fat body *UPD2* reduces adult body size by inhibiting the release of DILP2 from IPCs (Géminard et al., 2009; Wright et al., 2011; Rajan and Perrimon, 2012). On the other hand, sNPF-dependent increase in food consumption and body size is related to the effect of sNPF on insulin secretion as sNPF regulates the release of DILPs from IPCs (Lee K.S. et al., 2008).

Several microRNAs (miRNAs) have been also reported as critical regulators of ILP gene expression. For example, *miR-14*-mutants have a reduced lifespan with increased levels of TAG and DAG and an enlarged LDs, as well as decreased *DILP3* and *DILP5* expression, suggesting that miR-14 serves as a critical regulatory factor of lipid metabolism by down-regulating TAG and DAG synthesis (Xu et al., 2003). Additionally, specific down-regulation of *miR-14* in IPCs of the adult *Drosophila* brain increased lipid storage, whereas down-regulation in the fat body had no effect on fat stores (Varghese et al., 2010). miR-14 was found to regulate insulin metabolism through its direct target, *sugarbabe*, which encodes a predicted zinc finger protein that negatively regulates expression of several ILP genes, including *ILP3* and *ILP5* (Varghese et al., 2010). Thus, miR-14 exerts its effect on lipid storage indirectly through inhibition of an inhibitor of ILP expression. By contrast, *miR-278*-mutants were found to possess significantly reduced TAG levels, indicating that they induce lipogenesis (Teleman et al., 2006). Indeed, brain ILPs and trehalose levels increases in *miR-278*-mutants, suggesting that miR-278 interferes with the insulin pathway, and the reduction of lipid stores in *miR-278*-mutants is an outcome of a direct action of miR-278 on brain ILPs (Teleman et al., 2006). Another miRNA, miR-277, was also found to target other ILP genes (*ILP7* and *ILP8*) in the regulation of lipid deposition and mobilization in the mosquito *A. aegypti* (Ling et al., 2017). Another miRNA, miR-33, which is derived from an intron in *SREBP*, regulates genes involved in fatty acid metabolism and insulin signaling (Gerin et al., 2010; Dávalos et al., 2011). A genetic screen aiming to identify the miRNAs leading to inhibition of body growth in *Drosophila* revealed that miR-9a also acts on insulin signaling and body growth by controlling the expression of *sNPF* (Suh et al., 2015). Thus, IPC-specific over-expression of *miR-9a* reduces the insulin signaling and body size, and loss of *miR-9a* enhances the level of sNPF (Suh et al., 2015).

Another interesting topic on ILPs is their interaction with the Store-Operated Calcium Entry system, the SOCE, which also leads to changes in the lipid metabolism. For example, chronic knockdown of *STIM* leads to hyperglycemia, impairment of insulin signaling in fat body tissue, and formation of larger LDs accompanied by up-regulation of *4EBP* and a decrease in phosphorylated AKT levels (Xu et al., 2019). In addition, loss of function of the three brain ILPs was not found to prevent the extra fat accumulation in these knockdown insects (Xu et al., 2019). In accordance with these data, the insulin-promoting gene, *CCHa2*, was upregulated, whereas insulin-inhibiting genes, *ImpL2* and *Lst*, were down-regulated upon *STIM* down-regulation (Xu et al., 2019). Notably, *CCHa2*, which is expressed in the larval fat body and gut, is induced in response to dietary glucose (Sano et al., ı2015). Thus, mutants that lack *CCHa2* or the *CCHa2R* (CCHa2 Receptor) exhibit reduced DILP2 secretion and *DILP5* expression (Sano et al., ı2015). Lst suppresses DILP2 secretion, and Lst deficiency leads to hyperinsulinemia, hypoglycemia, and excess adiposity (Alfa et al., 2015). These results suggest that obesity is an outcome of the *STIM*-knock down related insulin signaling impairment which interferes with other neuropeptides, such as CCHa2 and Lst. Notably, other yet unknown neuropeptides could be also involved in this interaction.

Insulin signaling could lead to different outcomes in larval and adult stages (Kannan and Fridell, 2013; Owusu-Ansah and Perrimon, 2014); this is likely to be related to the differences in the physiology and feeding behavior. Thus, genetic ablation of the IPCs in larval stages of *Drosophila* leads to retardation in development and an increase in carbohydrate levels in the hemolymph (Rulifson et al., 2002). However, IPC ablation in the adult *Drosophila* reduces fecundity, increases stored TAG and sugars, and lifespan (Broughton et al., 2005). The overall evidence obtained to date suggest that brain ILPs are primarily controlled by sNPF, ecdysone, and foxO in larval stages, whereas miRNAs, foxO, and UPD2 are the major regulatory molecules involved in the transcriptional control of ILP genes in the adult stage, at least in *Drosophila* (Kannan and Fridell, 2013).

### Diapause Hormone-Pheromone Biosynthesis Activating Neuropeptide (DH-PBAN)

Diapause is a developmental arrest to overcome seasonal challenges, such as winter and the absence of food, and can occur in any developmental stage depending on the species (Denlinger et al., 2005). Various insects, such as the silkworm *B. mori* and the cotton bollworm *H. armigera*, have been shown to possess a specific peptide called the Diapause Hormone (DH) to regulate the process of diapause (Hasegawa, 1957; Xu et al., 1995; Zhang et al., 2004b).

DH is produced by neurosecretory cells in the subesophageal ganglion and possesses three regions; the N-terminal region that facilitates binding of the hormone to the Diapause Hormone Receptor (DHR), the middle region with its duplicated amino acid structure for full potency, and the carboxy-terminal essential core structure for biological activity (Saito et al., 1994). The Arg^23^ and Leu^24^ in the carboxy-terminal core structure are essential for binding to the DHR, whereas Trp^19^ and Phe^20^ contribute to functional activity (Shen et al., 2018). Interestingly, the carboxy-terminal active peptide (24 amino acids) is homologous to the carboxy-terminus of Pheromone Biosynthesis Activating Neuropeptide (PBAN), which is involved in female sex pheromone biosynthesis; therefore, DH is encoded by a PBAN gene and has been named DH-PBAN (Sato et al., 1993; Table 1).

*B. mori* overwinters in embryonic diapause, whereas *H. armigera* overwinters in pupal diapause (Zhang et al., 2004a, b). In the silk moth, DH-PBAN is secreted from the mother’s subesophageal ganglion and induces diapause in the eggs (Yamashita, 1996). Interestingly, while DH-PBAN induces diapause in *B. mori* (Xu et al., 1995), it reactivates metabolism in pupae and terminates pupal diapause in *H. armigera* (Xu and Denlinger, 2003; Zhang et al., 2004a, b). Therefore, DH-PBAN activation may lead to different outcomes for diapause initiation or termination.

DH-PBAN has been shown to affect lipid metabolism. For example, lipid content of eggs is slightly elevated by DH-PBAN (Hasegawa and Yamashita, 1965), however, this effect is likely to be a secondary consequence of the hormone’s effect on carbohydrate metabolism. DH-PBAN has been also reported to regulate the expression of genes involved in lipid metabolism in *H. armigera* (Majerowicz and Gondim, 2013).

Recent studies have revealed new insights into the DH-PBAN pathway. Binding of DH-PBAN to DHR induces activation of ERK phosphorylation through the signal transducer G-protein-linked PLC, PKC, and PI_3_K pathways (Jiang et al., 2016). More specifically, DHR is coupled with PLC via Gαq protein, leading to the production of DAG and the second messenger IP_3_ (Jiang et al., 2016). DAG directly activates PKC, and IP_3__–_mediated release of calcium from endoplasmic reticulum to cytosol leads to an indirect activation of PKC (Litosch, 2015). It would be interesting to further examine the SOCE components in DH-PBAN-controlled diapausing events.

### Short Neuropeptide F (sNPF) and Neuropeptide F (NPF)

Peptides of the Short Neuropeptide F (sNPF) and Neuropeptide F (NPF) (NPF) family are widely distributed throughout the Arthropoda phylum. NPF is functional homolog of mammalian Orexigenic Neuropeptide Y; the sNPF system is conserved across protostomes, but is not present in vertebrates (Fadda et al., 2019). Both systems are involved in the coordination of feeding behavior and metabolism (Lee et al., 2004; Nässel and Wegener, 2011; Mirabeau and Joly, 2013). Both systems also share common structural features; however, they are evolutionary distinct. The sNPF is characterized by an “M/T/L/FRF(W)” amide, and the NPF by an “RXRF(Y)” amide carboxy-terminal motif (Fadda et al., 2019). Most of the studies on these peptides in relation to lipid metabolism are on the sNPF.

The first insect sNPF was reported from *L. decemlineata* (Spittaels et al., 1996), followed by the discovery of sNPFs from the locust *S. gregaria* (Schoofs et al., 2001) and the fruit fly (Lee et al., 2004). The precursor is around 281 amino acid in length and the amidated peptide consists of 6–19 amino acids and possesses a carboxy-terminal “RLRF” sequence (Wegener and Gorbashov, 2008; Fadda et al., 2019; Table 1). sNPFs bind to the receptors (sNPFR), which are also from the rhodopsin-like GPCRs superfamily, like other neuropeptide receptors.

sNPFs are typically expressed by brain lateral neurosecretory cells, as well as in the midgut (Reiher et al., 2011), hindgut (Caers et al., 2016), antennae, Malpighian tubules, and ovaries (Jiang et al., 2017; Table 1). In most species, multiple sNPF isoforms exist and are derived from a single peptide precursor. For example, the sNPF precursor generates four sNPF isoforms in *Drosophila* (Vanden Broeck, 2001; Baggerman et al., 2002), the tsetse fly, *Glossina morsitans morsitans* (Caers et al., 2016) and *B. dorsalis* (Jiang et al., 2017), three sNPF isoforms in *B. mori* (Yamanaka et al., 2008) and *A. aegypti* (Veenstra, 1999), two peptides in *L. decemlineata* (Spittaels et al., 1996) and a single peptide in *R. prolixus* (Ons et al., 2011).

The main function of sNPFs is to regulate feeding behavior (Lee et al., 2004; Dillen et al., 2013); however, they may also be involved in locomotor activity (Kahsai et al., 2010), circadian rhythm (Johard et al., 2009; Geo et al., 2019), appetitive olfactory behavior (Root et al., 2011; Jiang et al., 2017), sleep homeostasis (Chen et al., 2013) and release of other hormones such as ILPs and AKH (Nässel et al., 2008). Regarding their primary role, sNPFs promote food intake and feeding in *Drosophila*, therefore, they could be considered as hunger hormones (Lee et al., 2004; Root et al., 2011). Over-expression of *sNPF* produces larger flies (Lee et al., 2004). sNPF also induces feeding in *B. mori* (Nagata et al., 2011). Presence of sNPF during feeding and its absence in the diapausing stage in *L. decemlineata* also suggests a positive correlation between feeding and sNPF activity (Huybrechts et al., 2004). On the other hand, inhibitory effects of sNPFs on feeding have been demonstrated in *A. aegypti* (Nässel et al., 2008), *S. gregaria* (Dillen et al., 2013), and *Culex quinquefasciatus* (Christ et al., 2018). sNPF has been shown to inhibit the serotonin-induced peristaltic contractions and ion transport in the anterior stomach of *A. aegypti* larvae (Onken et al., 2004). sNPF also inhibits the release of digestive enzymes in the cockroach *Periplaneta americana*, indicating an inhibitory effect of sNPF on digestion (Mikani et al., 2012). The inhibitory effect on the digestive process might be one of the reasons for sNPF-reduced feeding behavior. Notably, the sNPF level in the antennal lobes drops following a blood meal, indicating an inhibition of odor-mediated host seeking behavior (Onken et al., 2004). In *S. gregaria*, RNAi-mediated silencing of the sNPF precursor leads to an increase in food intake (Dillen et al., 2013, 2014). These findings suggest that there is not an obvious correlation between sNPF and feeding in the class of insecta and that sNPF can act as a stimulatory or inhibitory factor on feeding.

sNPF also affects lipid metabolism, for example, down-regulation of *sNPF* reduces food intake (Lee et al., 2004), increases starvation sensitivity (Kahsai et al., 2010), and causes lean flies in *Drosophila* (Baumbach et al., 2014a). A recent study also revealed that knockdown of *sNPF* in circadian clock neurons reduced TAG level (Geo et al., 2019). In accordance with this, over-expression of *sNPF* causes hyperphagia and body fat accumulation in *Drosophila* adults (Baumbach et al., 2014a); this effect could be via the effect on ILPs. Thus, *sNPF* expression is up-regulated in starved flies, where insulin levels are low, leading to initiation of food search behavior (Root et al., 2011). The Mnb/Dyrk1a kinase (Minibrain/Dual-specificity Tyrosine Phosphorylation-regulated Kinase 1A), which is localized to *sNPF*-expressing neurons and activates foxO, was found to be the most pronounced and up-regulated gene after sNPF administration, further showing a connection between sNPF and lipolysis (Hong et al., 2012). In support of this, activation of Mnb/Dyrk1a kinase occurs through PKA and CREB which also induce lipolysis (Hong et al., 2012). *CREB* is up-regulated by its binding partner cAMP-regulated transcription co-activator (CRTC) to induce the expression of *sNPF*, resulting in an increased starvation resistance (Shen et al., 2016). Therefore, sNPF is a direct target of CREB and CRTC. On the other hand, sNPF has been demonstrated to stimulate the production of ILPs in larval and adult IPCs in *Drosophila* (Lee K.S. et al., 2008; Kapan et al., 2012). In this manner, sNPF activates extracellular activated receptor kinases in IPCs, which leads to production of insulin (Lee K.S. et al., 2008; Kapan et al., 2012). More specifically, the fat body of *sNPF*-mutant *Drosophila* had down-regulated *AKT* expression and nuclear-localized foxO, up-regulated translational inhibitor *4E-BP* and reduced cell size with elevated glucose levels (Lee K.S. et al., 2008). On the other hand, knockdown of *sNPF* in the dorsal lateral peptidergic neurons results in diminished *DILP2* and *DILP5* expression leading to increased starvation resistance and increased levels of carbohydrates and lipid (Lee K.S. et al., 2008). These findings are indeed contradictory to the proposed role of sNPF as a hunger signal in *Drosophila*, however, up-regulation of DILP genes by sNPF might relate more to regulating metabolism and growth, rather than feeding (Lin et al., 2019).

The first insect NPF identified from *Drosophila* consisted of 36 amino acids with a characteristic “RVRF” carboxy-terminal sequence (Brown et al., 1999; Table 1). The carboxy-terminal tyrosine residue in vertebrate NPYs is replaced with a phenylalanine residue; therefore, these peptides were designated as “NPF” (Maule et al., 1991). The NPF precursor is around 102 amino acid in length and the active amidated peptides typically consist of at least 28 amino acids and share the common “RXRF/Y” carboxy-terminal motif (Fadda et al., 2019; Table 1). NPF is localized in the midgut and brain in *Drosophila* (Brown et al., 1999; Lee et al., 2006). Similarly, NPF was detected in the midgut and the subesophageal ganglion in *A. aegypti* (Onken et al., 2004; Table 1).

The *Drosophila* NPF has been shown to be important for adult longevity, wakefulness and feeding behavior, modulation of odor-aroused appetitive behavior, and reproduction (Gendron et al., 2014; Chung et al., 2017; Harvanek et al., 2017). However, most of the studies focused on the activation of NPFs by sugars, in particular in *Drosophila* larvae. Interestingly, the causative agent for the NPF activation is not sugar ingestion itself, instead taste perception induces the *NPF* expression (Shen and Cai, 2001). Additionally, *NPF* expression was found to be high in young larvae, whereas it was low in older larvae that avoid food (Wu et al., 2003). The interaction between the NPF and sugar feeding may indicate that insulin might also have an effect on *NPF* expression. In a recent study, NPF has been shown to function downstream of insulin signaling to regulate feeding in *Drosophila* larvae (Fadda et al., 2019). NPF not only affects feeding, but also regulates food choice as opposed to sNPF (Wu et al., 2005a, b). In support of this, NPF induces feeding on lower quality or noxious foods in starved fruit flies, whereas NPF-induced feeding response toward noxious food is inhibited in satiated fruit flies (Wu et al., 2005b). Furthermore, the inhibitory effect was found to be insulin-related. In *S. gregaria*, injection of NPF increases food intake and weight, while silencing *NPF* decreases food intake and weight, suggesting a stimulatory role of NPF in feeding (Van Wielendaele et al., 2013). Similarly, knockdown of *NPF* resulted in a reduction of food intake and growth in *B. mori*, also suggesting a role for NPF as a positive regulator of feeding (Deng et al., 2014). A recent study indicated that the enzymatic cofactor tetrahydrobiopterin (also known as BH4) inhibits NPF release, and might be one of the signals that transmit the message of energy status from the fat body to the brain (Kim et al., 2017).

Evidence on the involvement of NPF in lipid metabolism is limited. Activation of the *NPF*-expressing neurons has been shown to decrease TAG levels in adult *Drosophila* (Chung et al., 2017). Another study revealed that adult male fruit flies exposed to female sex pheromone displayed reduced TAG levels in parallel to an increase in the levels of NPF in the brain, however, inhibition of *NPF*-expressing neuron activity and down-regulation of *NPF* reverses these effects (Gendron et al., 2014). Based on the limited evidence in these studies and, one could say that NPF activation leads to reduced TAG levels.

### Allatostatin-A (AstA)

Allatostatin-A (AstA) is mainly expressed in the brain and gut (Veenstra et al., 2008; Hentze et al., 2015) and was originally reported to be involved in the inhibition of JH synthesis in the cockroach *Diploptera punctata* (Yoon and Stay, 1995). However, subsequent studies revealed that AstA does not regulate JH in all insects and is involved in the inhibition of the starvation-induced feeding behavior in *Drosophila* (Hergarden et al., 2012; Hentze et al., 2015; Chen et al., 2016). Furthermore, activation of *NPF*-expressing neurons suppresses the inhibitory influence of AstA neuron activation on feeding, leading to increased feeding (Hergarden et al., 2012).

AstA regulates AKH and ILPs through its galanin-like receptor “DAR2 (*Drosophila* Allatostatin Receptor 2” that is expressed in AKH- and ILP-producing cells (Hentze et al., 2015; Nässel and Vanden Broeck, 2015; Table 1). Thus, both insulin and AKH signaling are stimulated by AstA via DAR2; silencing *DAR2* reduced both ILPs and AKH and increased starvation resistance (Buch et al., 2008; Nässel and Vanden Broeck, 2015; Hentze et al., 2015). Furthermore, Drosophila lacking *AstA* accumulate high lipid levels, indicating that reduced AKH and ILP signaling also promotes lipid accumulation in the fat body (Hentze et al., 2015). Notably, *AstA* and *DAR2* expression differ according to the diet and gender (Hentze et al., 2015). These data suggest that AstA regulates the balance between AKH and ILPs and is important for the maintenance of nutrient homeostasis in Drosophila.

### Corazonin (Crz)

Corazonin (Crz) is a neuropeptide present as a 154 amino acid precursor and 11 amino acid of mature peptide (Choi et al., 2005; Table 1). It is produced by the brain lateral neurosecretory cells (Duan-Şahbaz and Ýyison, 2018). Crz was originally isolated as a cardioactive factor in *P. americana* (Veenstra, 1989). Subsequent studies revealed that it is also involved in the regulation of the ecdysis initiation (Kim et al., 2004), melanization (Žitòan and Daubnerová, 2016), stress responses (Kubrak et al., 2016), sperm transfer and copulation (Tayler et al., 2012), social behavior and caste identity (Gospocic et al., 2017), and ethanol sedation (Sha et al., 2014).

Crz is evolutionarily related to AKH (Veenstra, 1994) and expression of *Crz* is reduced by 50% in *AKH*-mutants (Choi et al., 2008). Additionally, ablation of *Crz* decreases trehalose levels (Choi et al., 2008; Lee K.S. et al., 2008). In this manner, Crz might modulate AKH-cell functions through neuronal pathways or trehalose levels by acting as a hormone on adipocytes and AKH-producing cells (Choi et al., 2008). On the other hand, activation of Crz-producing neurons leads to increased food uptake in adult flies (Lee G. et al., 2008; Zhao et al., 2010). The *Drosophila* Crz Receptor (CrzR) is also related to the family of AKH receptors; however, CrzR is highly selective for Crz (Park et al., 2002; Kim et al., 2004). Notably, intermediates similar to both AKHR and CrzR have been reported for a neuropeptide named as ACP (AKH/Corazonin-related Peptide) that is also structurally intermediate between AKH and Crz (Hansen et al., 2010). *Crz* is expressed primarily by dorsolateral peptidergic neurons, as well as abdominal ganglia, whereas *CrzR* is expressed in adult salivary glands and fat body (Sha et al., 2014; Kubrak et al., 2016).

Crz has been shown to affect lipid and carbohydrate metabolism (Kapan et al., 2012; Kubrak et al., 2016; Gáliková et al., 2017). Knockdown of *CrzR* in the fat body leads to a decrease in TAG levels and food intake, and an upregulation of *bmm* only after starvation (Kubrak et al., 2016). However, ablation or inactivation of the *Crz*-expressing dorsolateral peptidergic neurons in the brain resulted in increased TAG levels, suggesting that Crz decreases energy reserves (Zhao et al., 2010). Similarly, knockdown of *Crz* in dorsolateral peptidergic neurons in the *Drosophila* brain increases TAG levels and circulating glucose (Kapan et al., 2012). Knockdown of *CrzR* in the fat body or in the periphery also increases glucose levels in the hemolymph, but only in response to starvation as fed-flies did not show any altered levels of circulating glucose upon *CrzR* silencing (Kubrak et al., 2016). On the other hand, production of brain ILPs varies in response to diminished CrzR. For example, *ILP5* expression increased only in fed-flies, whereas *ILP3* expression decreased both in fed and starved flies, but *ILP2* was not found to change (Kubrak et al., 2016). Notably, expression of all three ILP genes decreased upon a longer period of starvation (36 h), but there was no significant change at the peptide level upon *CrzR* knockdown (Kubrak et al., 2016). These findings suggest that the effect on carbohydrate metabolism is mediated by Crz signaling to the periphery and this effect is stronger during stress conditions, such as starvation. Additionally, fat-body-derived humoral signals are affected by Crz activation of adipocytes. As a final point, the effect of Crz on lipid metabolism is likely to be indirect and occurs through insulin or another signaling system.

### Leucokinin (Lk)

Leucokinin (Lk) is a myotropic neuropeptide and initially identified as a neurohormone that plays an important role in water and ion homeostasis by regulating fluid secretion in the Malpighian tubules and hindgut motility (Holman and Cook, 1983; Schoofs et al., 1992; Table 1). Lk is also involved in meal size regulation, feeding, metabolic rate, post-feeding physiology and behavior, regulation of stress, water homeostasis, locomotor activity and sleep (Al-Anzi et al., 2010; Zandawala et al., 2018; Yurgel et al., 2019). The first Lk was isolated from the cockroach *Leucophaea maderae* (Holman et al., 1986), followed by identification of other Lks from *L. migratoria* (Schoofs et al., 1992), *A. aegypti* (Veenstra, 1994) and *Culex salinarius* (Hayes et al., 1989). The Lks consist of about 160 amino acids and the active peptides vary from 6 to 15 amino acids in length and are characterized by a carboxy-terminal pentapeptide motif “FXXWG” amide, which is essential for biological activity (Radford et al., 2002; Table 1).

*Drosophila* has a single Lk gene encoding the longest known leucokinin, “Drosokinin” and a Leucokinin Receptor (LkR) has been also identified (Radford et al., 2002). There is no known mammalian counterpart for Lk, but LkR is homologous to the vertebrate “Tachykinin Receptor, TkR” (Radford et al., 2002). *Lk* and *LkR* are expressed in small subsets of neurons in the brain, IPCs and ventral ganglia, and *LkR* is also expressed in the foregut, hindgut, Malpighian tubules and genital tracts (Radford et al., 2002; Al-Anzi et al., 2010; Zandawala et al., 2018).

During feeding, *Lk-* and *LkR*-mutant adult flies consume larger meals, but exhibit reduced long-term food intake (Al-Anzi et al., 2010; Liu Y. et al., 2015; Zandawala et al., 2018). The activity of Lk neurons is modulated by feeding with reduced activity in response to glucose and increased activity under starvation conditions (Yurgel et al., 2019). Thus, the effect of Lks on lipid metabolism is indirect and likely occurs through insulin signaling. In support of this, *Lk-* and *LkR*-mutants or flies with targeted knockdown of *LkR* in IPCs displayed altered expression of ILP genes, increased *DILP2* and *DILP3* in IPCs, and increased starvation resistance, suggesting a role for Lk in regulation of insulin signaling (Zandawala et al., 2018). Based on this data, Lk might act as a starvation-induced lipolytic agent; however, no study has examined this to date.

### CCHamide-2 (CCHa2)

CCHamide-2 (CCHa2) is also a typical orexigenic brain-gut peptide without a known counterpart in mammals (Lin et al., 2019). *CCHa2* is expressed in the brain; as well as fat body and the midgut (Ren et al., 2015; Sano et al., 2015; Table 1). *CCHa2* expression decreases in response to starvation and levels can be rescued by feeding on glucose or yeast (Sano et al., 2015). In accordance with this, *Drosophila* larvae and adult flies lacking CCHa2 show reduced feeding activity, indicating the stimulatory effect of CCHa2 on feeding (Ren et al., 2015).

CCHa2 binds to its receptor, CCHamide-2 Receptor (CCHa2R), in IPCs to promote insulin signaling (Ren et al., 2015; Sano et al., 2015). Thus, disruption of CCHa2R was found to reduce ILP concentrations and larval growth, which is consistent with late pupariation observed in the *CCHa2*-mutants (Sano et al., 2015). Gáliková et al. (2017) suggested that the repression of the central ILPs by AKH might be at least partially mediated by the CCHa2. Overall, the effect of CCHa2 on lipid metabolism might be lipogenic; however, this has not been specifically reported. Nevertheless, the effect occurs indirectly through interaction with insulin signaling.

### Tachykinins (Tk)

Gut peptide hormones play crucial roles in systemic lipid homeostasis (Song et al., 2014). The most abundant gut hormone is tachykinin (Tk), which produces six mature peptides (Tk1-Tk6) in Drosophila (Veenstra et al., 2008; Reiher et al., 2011; Table 1). Many other invertebrates, and even humans have also Tks. Notably, Drosophila Tks are also produced in the central nervous system, and brain Tks are involved in locomotor activity and olfactory responses (Winther et al., 2006; Birse et al., 2011; Reiher et al., 2011; Table 1). However, only gut Tks have been specifically shown to repress intestinal lipogenesis, which occurs via the G-protein-coupled Tachykinin Receptor (*TkR*) that is also expressed in gut (Song et al., 2014). TAG levels were dramatically increased in the midgut, fat body and hemolymph in the absence of gut Tks (Song et al., 2014). In accordance with this observation, genes encoding the intestinal lipase Magro, and the two key enzymes of lipogenesis, FAS and ACC, were all found to be up-regulated when Tk production was reduced, also confirming that Tk deficiency promotes midgut lipogenesis (Song et al., 2014). Notably, the suppressive role of Tks on lipogenesis occurs through repression of SREBP, a transcription factor that triggers lipogenesis. On the other hand, expression of the foxO target genes, *4EBP* and *InR*, in the midgut was not affected by removal of Tks (Song et al., 2014). However, knockdown of *TkR* in *Drosophila* induces expression of *ILP2* and *ILP3* in fed flies, and *ILP2* in starved flies, whereas expression of *ILP3* was reduced in starved flies (Birse et al., 2011) suggesting that gut Tks may affect insulin signaling in the midgut.

### Cytokines (Adipokines)

Fat body adipocytes secrete protein hormones termed cytokines (also known as adipokines). One hormone in this group is the leptin-like cytokine, the UPD2 (Rajan and Perrimon, 2012; Table 1). UPD2 indeed acts on brain IPCs, which release ILPs under the control of the brain gamma-aminobutyric acid (GABA) (Nässel and Vanden Broeck, 2016). IPCs also possess metabotropic GABA receptors (Enell et al., 2010) and are hyperpolarized by GABA (Rajan and Perrimon, 2012). This GABAergic inhibition can be disengaged by UPD2 (Rajan and Perrimon, 2012). Notably, specific perturbation of UPD2 function in the fat body alters energy metabolism and inhibits development (Rajan and Perrimon, 2012). UPD2 release from the fat body is triggered by elevated levels of lipid or carbohydrate in the hemolymph, thus, UPD2 senses the fed state and regulates secretion of brain ILPs (Rajan and Perrimon, 2012). Therefore, in the fed state, circulating UPD2 binds to its transmembrane receptor, “domeless,” which activates the JAK/STAT signaling in the GABAergic neurons, blocking GABA release and diminishing IPC hyperpolarization resulting in secretion of ILPs (Brown et al., 2001; Rajan and Perrimon, 2012; Lin et al., 2019). Thus, flies with *UPD2* knockdown in fat body exhibited increased ILP accumulation in the brain under a fed state (Rajan and Perrimon, 2012). By contrast, IPCs are inhibited by GABAergic neurons that hyperpolarize IPCs in the starved state, thus, *UPD2* is down-regulated in starving adults. In accordance with this, fat body-specific knockdown of *UPD2* resulted in hyperglycemic, lean flies and larvae with considerably reduced TAG and increased circulating sugar levels. It is noteworthy to mention that *UPD2*-mutant larvae had dramatic accumulation of LDs in oenocytes, suggesting an opposite function for oenocytes and adipocytes in lipid metabolism (Rajan and Perrimon, 2012; Lin et al., 2019). Thus, UPD2 suppresses stored fat breakdown in oenocytes during starvation (Rajan and Perrimon, 2012; Lin et al., 2019). *UPD2* was found to be downregulated in the fat body after *CrzR*-knockdown, suggesting UPD2 also serves as a messenger between the fat body and the brain by acting on brain ILPs. On the other hand, Unpaired 1 (UPD1), another fly leptin-like peptide, fulfills the roles of UPD2 upon *UPD2* knockdown in adults (Beshel et al., 2017). Unlike UPD2, which is secreted from fat body, UPD1 is produced by a small cluster of neurons in the brain (Table 1).

Another peptide belonging to this group is the Adiponectin (Adipo), which regulates glucose levels and fatty acid breakdown in mammals. No obvious Adipo homolog has been identified in *Drosophila*; however, an Adiponectin Receptor (AdipoR) with high homology to the human Adiponectin Receptor 1 has been discovered (Kwak et al., 2013; Laws et al., 2015). The *Drosophila AdipoR* is expressed in the IPCs of larval and adult brains (Kwak et al., 2013). Inhibition of AdipoR leads to elevated sugar levels in the hemolymph, TAG levels in whole body, and ILP2 accumulation in IPCs (Kwak et al., 2013). In contrast, the level of circulating ILP2 and insulin signaling were reduced in the fat body (Kwak et al., 2013). A subsequent study revealed the requirement of AdipoR in germline stem cell maintenance in the *Drosophila* ovary (Laws et al., 2015). In brief, AdipoR modulates insulin secretion and lipid metabolism. Additionally, Adipo signaling is intrinsically required for stem cell maintenance independently of insulin signaling (Laws et al., 2015).

### Limostatin (Lst)

Limostatin (Lst) is a known suppressor of insulin production and expressed by AKH-producing neurons in the corpora cardiaca and fat body, in particular during starvation (Alfa et al., 2015; Table 1). Thus, *Lst*-mutant flies were found to be hypoglycemic with increased levels of *DILP2*, *DILP3*, and *DILP5* mRNA (Alfa et al., 2015). Additionally, Lst levels decreased in an AKH-deficient background; however, *AKH* over-expression did not significantly increase *Lst* expression. In brief, Lst leads to lipolysis; however, this effect is an outcome of insulin suppression.

## Role of Peptide Hormones in Lipid Metabolism-Related Biological Events

In this section, the peptide hormones involved in lipid metabolism-related biological events together with their role(s) in these events are examined.

### Reproduction

Lipids plays a critical role in reproductive physiology and are mobilized as the major metabolic source during reproduction (Hansen et al., 2013), therefore, peptide hormones involved in lipid metabolism have also essential roles in reproduction. AKH, ILPs, and sNPF are the major peptide hormones involved in lipid metabolism in relation to reproduction.

Role of AKH signaling in insect reproduction has been studied in several insects. For example, AKHR knockdown led to obese females incapable of utilizing their lipid reserves during pregnancy for milk production *G. m. morsitans* (Attardo et al., 2012). Such silencing also resulted in delayed oocyte development with a reduction of 20% in fecundity (Attardo et al., 2012). Additionally, knockdown of the *AKH*/*AKHR*-mediated lipolytic system affected larvigenesis as suppression of *AKHR* expression lowered production (offspring per female). AKH also inhibits egg-laying indirectly in *G. bimaculatus* due to the reduction in fat body lipid stores by AKH during vitellogenesis (Lorenz, 2003). In *B. dorsalis*, *AKHR* silencing was found to lower lipolytic activity, delay oocyte maturation, and reduce fecundity (Hou et al., 2017). The inability of fat body to accumulate adequate nutrient reserves after AKH exposure has been also shown in the locust, *S. gregaria* (Gokuldas et al., 1988), and the mosquito, *A. aegypti* (Ziegler, 1997). The majority of the stored lipid in the oocytes is TAG and any failure to TAG accumulation and mobilization would affect fecundity and oocyte development, therefore, the increased rate of lipolysis might negatively affect reproduction (Lu J et al., 2018b). Notably, AKH also affects sexual courtship activity, as was shown in *B. dorsalis* (Hou et al., 2017). There are also reports indicating no noticeable effects of AKH on reproduction as genetic manipulation of *AKH* in adult *Drosophila* flies did not cause any negative outcome in the reproductive capabilities and courtship behavior of flies (Lee and Park, 2004).

ILPs have been also shown to affect reproduction. CRISPR/Cas9-mediated depletion of *ILP7* and *ILP8* leads to reproductive defects related to lipid homeostasis and ovarian development (Ling et al., 2017), and ILP7 is involved in egg-laying behavior (Yang et al., 2008). Fecundity was found to be reduced in *Drosophila* mutants lacking *ILP2* (Grönke et al., 2010). In a recent study, knockdown of insulin signaling genes *Chico*, *TOR* and *Slimfast*, a membrane transporter of amino acids that is involved in Target of Rapamycin Complex 1 (TORC1) signaling, was found to reduce the number of ootheca in *B. germanica* (Li et al., 2019). In another recent study, the c-Jun N-Terminal Kinase (JNK)-initiated insulin-myc signaling loop was shown to promote mitochondrial respiration and biogenesis in *Drosophila* ovary, suggesting the insulin-myc signaling is important for mitochondrial biogenesis in the ovary (Wang et al., 2019). A miRNA acting on insulin signaling, miR-277, has also been shown to be important in the reproduction of the mosquito *A. aegypti* as CRISPR/Cas9 deletion of *miR-277* led to failures in ovary development (Ling et al., 2017).

NPF and sNPF also exhibit several effects on insect reproduction. In locusts (Cerstiaens et al., 1999; Schoofs et al., 2001) and the fruit fly (Mertens et al., 2002), sNPF stimulates ovarian development (De Loof et al., 2001). On the other hand, the level of NPF was been found to be elevated in male fruit flies exposed to the sex pheromone of females, while TAG levels decreased (Gendron et al., 2014; Harvanek et al., 2017). However, these effects may be non-specific and do not have to be due to direct interactions with reproductive physiology.

### Flight

Many insects, in particular long distance flying insects, use lipids as the primary fuel for flight (Weis-Fogh, 1952). In this regard, AKH is a main determinant of successful energy demand (Ziegler and Schulz, 1986). During the first few minutes of flight, octopamine is released, inducing the first release of DAG from the fat body (Orchard et al., 1993), however, the subsequent, more prolonged phase of TAG mobilization occurs through the action of AKHs (Arrese and Soulages, 2010). As a result, the concentration of DAG in the hemolymph increases and constitutes the principal fuel for flight.

The effect of AKH on insect flight has been mostly studied in two locusts such as, *L. migratoria* and *S. gregaria*, and a moth, *M. sexta* (Van der Horst and Ryan, 2012). AKH peptides originate from pre-prohormones that are translated from different mRNAs and eventually enzymatically processed. Binding of the AKHs to their plasma membrane GPCRs on the fat body cells is the primary step in the induction of signal transduction events that lead to mobilization of lipids to be used by muscles as a fuel for flight (Vroemen et al., 1998). Such events require involvement of various other molecules, such as cAMP, PKA and IP_3_ (Van Marrewijk et al., 1996; Vroemen et al., 1997), lipases (Arrese and Wells, 1994; Ogoyi et al., 1998), lipophorins (Izumi et al., 1987; Van der Horst and Rodenburg, 2010), fatty acid binding proteins (Haunerland and Chisholm, 1990), and calcium ions (Van Marrewijk et al., 1991).

### Diapause

Lipid reserves are the most important resources for insects to meet energy demand during the dormancy state known as diapause (Hahn and Denlinger, 2011). Insects accumulate lipid reserves prior to diapause and a failure to accumulate adequate amounts of lipids leads to incomplete diapause and possibly death (Toprak et al., 2014b).

AKHs do not contribute to diapause-associated alterations in metabolism (Hahn and Denlinger, 2011). AKH is produced in response to AMPK, which leads to the release of DAG into hemolymph from TAG stores in the fat body via a cyclic cAMP and calcium signaling cascade during diapause maintenance (Sinclair and Marshall, 2018). For example, AKH has been shown to release approximately twice as much lipid into the hemolymph in diapausing adult females of the firebug *Pyrrhocoris apterus* versus the non-diapausing counterparts, suggesting diapausing adults have greater sensitivity to lipid-mobilization by AKH (Socha and Kodrik, 1999).

Insulin signaling also plays an important role in the regulation of diapause (Sim and Denlinger, 2008). This is not surprising as insulin is central to energy storage and suppresses the lipolytic action and nuclear translocation of foxO (Baker and Thummel, 2007). The lipid accumulating effect of insulin associated with adult diapause has been shown in *Drosophila* (Tatar and Yin, 2001), and *C. pipiens* (Sim and Denlinger, 2008). Additionally, silencing *InR* in non-diapausing females inhibits ovary development, which simulates the diapause state (Sim and Denlinger, 2008). As expected, the insulin effect occurs mainly during feeding, therefore, before the initiation of the diapause. For example, adult females of *C. pipiens* increase feeding with sugar instead of blood in the prediapause period and accumulate much greater lipid reserves compared to non-diapausing counterparts (Robich and Denlinger, 2005). In this manner, specific ILPs, such as ILP1, contribute to the diapause regulation-related lipid accumulation in *C. pipiens* (Sim and Denlinger, 2009). Notably, JH and ecdysone interfere with insulin signaling in terminating diapause (Denlinger et al., 2005).

NPF and sNPF might also affect lipid metabolism-related diapause regulation as they are involved in feeding behavior, nutritional homeostasis, and insulin signaling (Brown et al., 1999; Wu et al., 2003; Huybrechts et al., 2004). For example, overexpression of *NPF* leads to prolonged feeding in *Drosophila* larvae (Wu et al., 2003; Chung et al., 2017), therefore, *NPF*-mutant larvae feed less. This induces insulin signaling and affects lipid sources permitting preparation for diapause (Fadda et al., 2019). As mentioned before, sNPF is also involved in feeding behavior. In Colorado potato beetles, feeding adults were found to possess sNPF; however, diapausing beetles lack sNPF (Huybrechts et al., 2004). The authors speculated that sNPF could play a role in the adult diapause process and possibly contribute to prediapause shifts in feeding behavior associated with lipid accumulation (Huybrechts et al., 2004).

DH/PBAN is another peptide hormone regulating diapause, in particular during embryonic diapause in several insects (Yamashita, 1996). The best known example for the involvement of DH/PBAN in diapause is the one that occurs in *B. mori* (Sato et al., 1993; Xu et al., 1995). The DH is produced by female adults during summer and induces diapause in developing eggs that would otherwise hatch and begin developing during the unfavorable autumn and winter months (Klowden, 2007). The induction of diapause by DH occurs through the stimulation of trehalase activity in the developing embryos, which leads to generation of glycogen and eventually glycerol and sorbitol which are necessary for diapause in these eggs (Su et al., 1994). The decline in sorbitol by the end of diapause leads to development of the embryo (Horie et al., 2000). Other molecules, such as dopamine and ecdysteroids, might also affect the embryonic diapause (Noguchi and Hayakawa, 2001; Denlinger, 2002).

### Starvation

Lipids are the primary fuel consumed during starvation stress (Marron et al., 2003). The most important peptide to mobilize lipid during starvation is AKH as starvation induces AKH release into the hemolymph to signal hunger (Kim and Rulifson, 2004). Under fasting conditions, AKH induces utilization of stored energy by stimulating lipolysis, glycogenolysis and trehalose release through activation of cAMP signaling in the fat body (Kim and Rulifson, 2004; Bharucha et al., 2008). For example, injection of AKH into adult insects, such *L. migratoria* and *M. sexta*, stimulates the formation of DAG (Gäde and Beenakkers, 1977; Shapiro and Law, 1983). In *B. dorsalis*, *AKHR* was found to be up-regulated significantly upon starvation (Hou et al., 2017). In addition, knockdown of *AKHR* resulted in high levels of whole body lipids (obesity) at death, indicating an inability to mobilize lipid reserves during starvation (Attardo et al., 2012; Choi et al., 2015). This is likely due to the inability of flies to utilize lipid stores under starvation conditions (Grönke et al., 2007). In line with this, starvation was found to significantly induce the expression of *AKH* and *AKHR* also in the brown planthopper, *Nilaparvata lugens* (Lu X et al., 2018a). Additionally, *AKHR* silencing decreased DAG levels in the hemolymph and increased TAG levels in the fat body, whereas AKH injection led to a critical accumulation of DAG in the hemolymph and a severe reduction of TAG content in the fat body. In addition, knockdown of *AKHR* resulted in prolonged lifespan and high levels of whole-body TAG, indicating an inability to mobilize TAG reserves during starvation. This is also similar to that reported for tsetse fly (Attardo et al., 2012). It is noteworthy that increased DAG levels in hemolymph during starvation could be independent of AKH activity as other lipolytic factors, such as bmm or carbohydrate metabolism (trehalose levels), could affect the rate of lipolysis (Heier and Kühnlein, 2018; Zhou et al., 2018).

*Drosophila* has been proposed as a good model to study the biochemical background of starvation (Gibbs and Reynolds, 2012). Starvation resistance is linked to lipid content in several *Drosophila* species (van Herrewege and David, 1997; Bharathi et al., 2003). Lipids stored in the fat body of *Drosophila* are consumed rapidly upon starvation (Zinke et al., 1999; Lee and Park, 2004). Various proteins, such as LSD1 and bmm, are activated by AKH-dependent phosphorylation to initiate lipolysis to overcome the starvation stress (Canavoso and Wells, 2001; Grönke et al., 2007). Other lipolytic agents, such as the Hormone-Sensitive Lipase (HSL), is also likely to be involved in starvation-induced lipolysis as the HSL has been shown to be translocated to LDs during starvation in *Drosophila* (Kühnlein, 2012). The role of AKH in HSL secretion is a gray area and requires further studies. On the other hand, AKH has been shown to act as a metabolic stimulator causing hyperlipemia, an abnormally high concentration of lipids in the hemolymph (Lee and Park, 2004). Furthermore, starved flies devoid of AKH neurons lack starvation-induced hyperactivity and displayed strong resistance to starvation-induced death with a longer life span (Gáliková et al., 2015; Sajwan et al., 2015; Zemanová et al., 2016). These mutants were also not able to mobilize lipids efficiently and, therefore, do not utilize these reserves rapidly. In another study, Mochanová et al. (2018) reported that *AKH*-mutant females were more resistant to starvation with a longer life span compared to males. On the other hand, absence of AKH has been also shown to increase survival rate during starvation (Gáliková et al., 2015; Sajwan et al., 2015; Zemanová et al., 2016). However, knock down of the SOCE molecule, *STIM* that is involved in calcium transport leads into reduced AKH levels and life span, and abnormal lipid mobilization profile under starvation (Xu et al., 2019).

The interaction between the AKH and LKB1-SIK3 signaling and HDAC4 localization has been examined in terms of the starvation response in *Drosophila* (Choi et al., 2015). As already mentioned, PKA inhibits SIK3 via phosphorylation, which leads to the translocation of HDAC4 from the cytosol into the nucleus to activate foxO, resulting in lipolysis (Figure 1). In this manner, fasting inhibits or reduces the kinase activity of LKB1 on SIK3, and induces HDAC4 nuclear localization, which leads to foxO activation and up-regulation of *bmm* (Choi et al., 2015). In accordance with these findings, SIK3 Thr^196^ phosphorylation by LKB1 has been found to be reduced during fasting and when *AKH* was over-expressed compared to that in feeding conditions. Furthermore, HDAC4 was found to be localized to both cytoplasm and nuclei in *AKHR*-mutant larvae fasting for short periods (4 h); however, HDAC4 accumulated only in the nuclei of the fat body cells in mutants fasting for prolonged periods (∼10 hr) (Choi et al., 2015). This finding indicates the presence of mechanisms for HDAC4 localization during prolonged fasting, which are independent of AKH signaling. Interestingly, continuous production of active SIK3 blocked the prolonged fasting-induced nuclear localization of HDAC4 (Choi et al., 2015). Nevertheless, AKH plays a critical role in the localization of HDAC4 in fasting, in particular for shorter periods. Notably, these events work in parallel to reduce insulin signaling.

Insulin is another factor affecting starvation. Disruption of the insulin signaling promotes lipid accumulation and increases resistance to starvation (Clancy et al., 2001; Broughton et al., 2005). Insulin secretion is elevated in response to feeding and typically decreases during starvation (Britton et al., 2002; Ikeya et al., 2002; Géminard et al., 2009). A recent study in *Drosophila* described insulin as an orexigenic hormone during short periods of starvation (Sudhakar et al., 2020). InR activity is also reduced following starvation (Britton et al., 2002). Similarly, *DILP3* and *DILP5* are down-regulated during starvation (Ikeya et al., 2002). However, Chatterjee et al. (2014) demonstrated that insulin activation is specifically required in oenocytes during starvation to maintain starvation resistance. Thus, the fat body-derived ILP6 induces lipid uptake in oenocytes, promotes lipid turnover during fasting and increases starvation tolerance in fasting adult flies (Chatterjee et al., 2014). Notably, ILP6 resembles IGFs and suppresses brain ILPs. Furthermore, silencing of *ILP6* or inhibition of the insulin activity in oenocytes reduces starvation-induced accumulation of LDs, therefore, new lipid synthesis in oenocytes, and induced-sensitivity to starvation, indicating insulin signaling in oenocytes is crucial to maintain starvation resistance (Chatterjee et al., 2014). Interestingly, overexpression of *DILP6* in the fat body and gut did not induce starvation tolerance, but rather increased starvation sensitivity (Chatterjee et al., 2014). As such, starvation tolerance significantly decreased when *DILP6* expression was reduced in the fat body (Chatterjee et al., 2014). These findings are in accordance with the proposed analogy between oenocytes and mammalian hepatocytes (Gutierrez et al., 2007; Martins and Ramalho-Ortigao, 2012) and support the notion that oenocytes play a central role in the metabolic adaptation to starvation (Chatterjee et al., 2014). On the other hand, starvation induces a significant increase in the number and size of LDs in adult oenocytes as starvation also induces TAG levels in the mammalian liver (Ohama et al., 1994). Similarly, knockdown of the insulin target “*PEPCK* (Phosphoenolpyruvate Carboxykinase)” impaired starvation-induced lipid uptake in oenocytes. Overall, the study by Chatterjee et al. (2014) suggests the presence of an oenocyte-specific insulin activity, which is critical for the mobilization of stored lipid under fasting conditions in oenocytes. In brief, the role of insulin signaling on lipid metabolism is different between adipocyte and oenocytes.

In terms of the biochemical background of insulin signaling on lipid metabolism, inhibition of the foxO transcription by insulin is a central phenomenon (Giannakou and Partridge, 2007), however, foxO was not detected in adult oenocytes (Chatterjee et al., 2014), suggesting the effect of insulin signaling in oenocytes might be foxO-independent. Nevertheless, starvation leads to a decline in PIP_3_ levels and dephosphorylation of AKT and translocation foxO to the nucleus in the fat body adipocytes. The decrease in ILPs during starvation leads to up-regulation of the insulin signaling target, 4EBP, encoded by *Thor*, and dephosphorylation of existing Thor protein (Gibbs and Reynolds, 2012). In parallel, decreases in insulin signaling stimulate the dephosphorylation and nuclear translocation of foxO (Jünger et al., 2003; Puig et al., 2003), therefore, phosphorylation of foxO decreases *Thor* expression, which occurs by feeding and increase in insulin signaling. On the other hand, starvation also triggers the activation of the CREB co-activator, CRTC, in *Drosophila* (Wang et al., 2008). *CRTC-* mutant flies have reduced glycogen and lipid stores and are sensitive to starvation (Wang et al., 2008). In line with this, the increase in insulin signaling inhibits CRTC activity during feeding through SIK2-mediated phosphorylation, leading to degradation of CRTC (Figure 4). CRTC was not phosphorylated during refeeding in flies with defective of insulin signaling (Wang et al., 2008). Furthermore, depletion of neuronal SIK2 increases CRTC activity and resistance to starvation. In line with these findings, foxO activity was found to be elevated in *CRTC-*mutant flies in parallel to the depletion of lipid and glycogen (Wang et al., 2008). CRTC indeed acts in parallel with foxO as CRTC is dephosphorylated and activated during starvation (Figure 2). In support of this, silencing *AKT* increases CRTC activity (Wang et al., 2008). Overall, CRTC enhances survival during starvation (Wang et al., 2008).

Leucokinin is also involved in the lipid metabolism-related starvation response. *Lk*-and *LkR-*mutant flies eat excessively after starvation, but do not eat more than normal flies when continuously supplied with food, suggesting that the mutants consume abnormally large meals, but at a reduced frequency (Al-Anzi et al., 2010). The effect of Lk on starvation response might occur through its interaction with insulin signaling. *Lk-* and *LkR-*mutants or flies with targeted knockdown of *LkR* in IPCs displayed increased *DILP2* and *DILP3* expression in IPCs and increased starvation resistance (Zandawala et al., 2018). Notably, Lk has been also reported to be involved in the modulation of starvation-dependent changes in sleep (Zandawala et al., 2018; Yurgel et al., 2019). Lk neurons in the lateral horn of the fly brain are required for starvation-induced sleep suppression and activity of these neurons increases under starvation conditions (Yurgel et al., 2019). In line with this, knockdown of *Lk* in Lk-expressing neurons was found to induce sleep during starvation (Yurgel et al., 2019). Additionally, LkR function in the IPCs is required for starvation-induced sleep suppression as silencing *LkR* in DILP2 neurons prevented starvation-induced sleep loss. These finding suggest that LkR is required in DILP2 neurons for starvation-induced sleep suppression (Yurgel et al., 2019).

NPF and sNPF may also affect lipid metabolism in relation to starvation. In various insects, production of these peptides is induced following starvation and decreases with feeding (Chung et al., 2017; Lin et al., 2019). Thus, sNPFR genes are up-regulated by starvation in fruit flies (Lee K.S. et al., 2008; Jiang et al., 2017), cockroaches (Mikani et al., 2012), and foraging honeybees (Ament et al., 2011). A recent study in *Drosophila* also revealed that knockdown of *sNPF* in circadian clock neurons reduced TAG level, starvation resistance and increased the starvation-mediated hyperactivity response after 24 h of starvation (Geo et al., 2019). Additionally, knock down of *sNPFR* expressed in IPCs was found to increase starvation resistance, but reduced the starvation-induced hyperactivity response after 24 h of starvation. On the other hand, *NPF*-mutant flies have been shown not to suppress sleep following prolonged starvation conditions, suggesting that NPF also acts as a hunger signal to keep the animal awake (Chung et al., 2017). Furthermore, activation of *NPF*-expressing neurons was found to decrease whole body TAG levels and increase food consumption and sensitivity to starvation conditions (Chung et al., 2017). The decrease in TAG levels by an increase in food consumption could be related to the activity of the leptin, UPD1. The UPD1 receptor domeless is expressed in *NPF*-expressing neurons, *Drosophila* domeless can be activated by human leptin, and feeding behavior is perturbed in flies lacking UPD1 (Rajan and Perrimon, 2012; Beshel and Zhong, 2013). Additionally, UPD1/domeless signaling suppresses NPF activity; and absence of this signaling leads to food overconsumption (Beshel et al., 2017). On the other hand, disruption of UPD2 in adipose tissue contributes to a reduction in body size (Rajan and Perrimon, 2012), indicating differences between the actions of the two domeless ligands (UPD1 and UPD2) (Beshel et al., 2017). It is noteworthy that there are contradictory findings on the reaction of NPF and sNPF toward starvation. *sNPFR* is down-regulated upon starvation in *B. mori* (Nagata et al., 2012), *Solenopsis invicta* (Chen and Pietrantonio, 2006) or *S. gregaria* (Dillen et al., 2013), however, *sNPF*/*sNPFR* expression increases transiently after feeding in these insects. Overall, sNPF positively regulates feeding in most species.

AstA is also involved in the starvation response as it inhibits starvation-induced feeding behavior, which leads to lipolysis (Hergarden et al., 2012; Hentze et al., 2015; Chen et al., 2016). Hergarden et al. (2012) indicated that AstA activation is likely to be an outcome, not a cause, of metabolic changes that induce the state of satiety. Other hormones, such as octopamine, could also affect lipid-metabolism-related starvation responses, in particular by affecting the release of peptide hormones, such as insulin (Li et al., 2016). However, such interactions are not included in the current review as octopamine is an amine hormone.

### Infections and Immunity

Infections or mutualistic interactions could alter lipid metabolism, therefore, immunity is also another factor affecting lipid homeostasis. Infections may lower whole body TAG levels according to the studies in Drosophila. For example, infection of *Drosophila* with *Listeria monocytogenes* leads to a decrease in both stored fats and glycogen (Chambers et al., 2012) and infections initiates host responses that lead to inhibition of TOR activity, which results in lipolysis in adipocytes (Lee et al., 2018). In accordance with this, microbe-free *Drosophila* flies possess elevated TAG levels compared to conventionally-reared counterparts (Wong et al., 2014). On the other hand, mutualistic bacteria, such as *Lactobacillus brevis* and *Acetobacter fabarum*, lower TAG levels in *Drosophila* (Sommer and Newell, 2019).

Several peptide hormones involved in insect lipid metabolism interfere with insect immunity. One example of this is AKH, which has been shown to activate the prophenoloxidase cascade (Goldsworthy et al., 2003; Mullen and Goldsworthy, 2006). In *L. migratoria*, phenoloxidase activity is induced more in response to laminarin when applied with AKH, compared to laminarin alone (Goldsworthy et al., 2002). In another study, the injection of a lipopolysaccharide from *Escherichia coli* with AKH resulted in the formation of a higher number of nodules compared to injection of the lipopolysaccharide only (Goldsworthy et al., 2003). The venom of the parasitoid *Habrobracon hebetor* up-regulated *AKH* expression in paralyzed adult females of *P. apterus* (Shaik et al., 2017). Furthermore, co-application of venom with AKH reduced paralysis compared to the application of venom only. Infection of *P. apterus* adults by the entomopathogenic nematode *Steinernema carpocapsae* up-regulated *AKH* (Ibrahim et al., 2017). These studies suggest that AKH is elevated upon infection and this increase can induce immune responses.

Insulin is another peptide hormone that interferes with infections and insect immunity (Galenza and Foley, 2019). For example, infection by *Mycobacterium marinum* leads to a decrease in lipid and glycogen stores in fruit flies by impairing insulin signaling through reduced AKT activation (Dionne et al., 2006). A previous study showed that the InR substrate *chico*-mutant homozygous and heterozygous flies have increased resistance against two pathogenic bacteria, the Gram-negative *Pseudomonas aeruginosa* and the Gram-positive *Enterococcus faecalis*; however, the mutants displayed a nearly threefold increase in survival, but no alteration in the expression of antimicrobial peptide genes upon infection (Libert et al., 2008). Interestingly, *Thor* was up-regulated twofold in *chico*-mutant homozygous flies upon infection. In line with this, Thor has been previously implicated in pathogen resistance of *Drosophila* (Bernal and Kimbrell, 2000). Libert et al. (2008) suggested that decreased insulin signaling in *chico*-mutant flies causes higher foxO activity, which leads to up-regulation of its target *Thor*, and improved survival of *Chico* mutants (Libert et al., 2008). *Chico*-mutant *Drosophila* flies (hypomorphic, but not null alleles) exhibit increased phenoloxidase activity and melanization response, and reduced phagocytosis in response to the insect pathogen *Photorhabdus luminescens* and non-pathogenic *Escherichia coli* (McCormack et al., 2016). Furthermore, the mutants contained lower pathogen titers in response to *P. luminescens* infection compared to controls, suggesting *chico*-mutants have increased resistance to infection. On the other hand, infected flies showed reduced transcript levels of antimicrobial peptide genes in the *chico*-mutants; however, *chico* mutation does not affect the survival upon bacterial infection which is in contrast to the findings by Libert et al. (2008). Notably, foxO could also induce expression of antimicrobial peptide genes in the fat body (Becker et al., 2010). *Chico* silencing in the fat body promotes the expression of the gene encoding the peptidoglycan receptor protein PGRP-SC2, but suppresses the expression of *PGRP-SB2*; both are important for development (Musselman et al., 2018a). These findings indicate a complex role for insulin activity in the host response that is highly context-dependent and varies for individual pathogens (Galenza and Foley, 2019). Nevertheless, chico plays an important role in the regulation of the antibacterial immune function. This could be related to decreased insulin signaling, which increases longevity, a common phenomenon in immunometabolism.

The gut hormone Tk could also interfere with insect midgut immunity, for example, microbial-derived acetate has been shown to induce *Tk* expression (Kamareddine et al., 2018). As Tk reduces insulin signaling and lipid storage (Birse et al., 2011; Song et al., 2014), it may be one of the reasons for the infection-related TAG decrease.

sNPF could be also involved in insect immunity as loss of sNPF signaling disrupts gut epithelial integrity and up-regulates anti-microbial peptide genes (Shen et al., 2016). By contrast, over-expression of *sNPF* has been found to dampen the gut immune response (Shen et al., 2016).

## Potential of Lipid Metabolism-Related Peptide Hormones in Pest Management

Pest control strategies targeting insect lipid metabolism has great potential due to the essential roles of lipids in insect biology and physiology. Various molecules targeting insect lipid metabolism have been developed and used already in the field as registered insecticides against various pests. In this manner, the most-important group is the lipid synthesis inhibitors, such as spiromesifen, spirodiclofen and spirotetramat (Nauen and Konanz, 2005; Nauen et al., 2008). Other groups of insecticides including JH analogs such as pyriproxifen (Fotouhi et al., 2015), chitin synthesis inhibitors such as hexaflumuron (Mirhaghparast et al., 2015), or synthetic pyrethroids (Balabanidou et al., 2016), organophosphates (Li et al., 2016) and neonicotinoids (Clements et al., 2020) have been also shown to impair directly or indirectly insect lipid metabolism. As this review focuses on the peptide hormones involved in insect lipid metabolism, this section is also restricted to the developments and potential of approaches targeting these hormones.

Strategies targeting peptides hormones involved in lipid metabolism in pest control is a developing area; therefore the progress at this stage is limited; however, there are promising findings. In this manner, efforts have focuses on the development of peptide hormone agonists/antagonists that have the potential of being replacements to chemical insecticides, or at least being used within integrated pest management programs (Fónagy, 2006). One promising candidate is AKH. The co-application of insecticides with AKH has been shown to increase their efficacy (Kodrík et al., 2015; Plavšin et al., 2015). In another study, co-application *S. carpocapsae* with AKH was found to enhance the mortality of the host *P. apterus* adults about 2.5 times within 24 h (Ibrahim et al., 2017). By contrast, firebugs with reduced expression of *AKHR* displayed significantly lower mortality (Ibrahim et al., 2017). The stimulatory effect of AKH on the efficacy of entomopathogenic fungi or bacteria has been also reported (Goldsworthy et al., 2005; Mullen and Goldsworthy, 2006). These studies all suggest that AKH has a potential for use with other pest control tools.

Another promising peptide hormone to be targeted is DH. Synthetic DH agonists/antagonists were examined in terms of their effect on the pupal diapause of the corn earworm, *Heliothis zea*. These studies revealed that DH agonists leads to an inhibition in the entrance into pupal diapause or a premature termination of diapause, while DH antagonists block the termination of diapause, suggesting DH analogs and antagonists are promising candidates for pest management by disrupting diapause (Zhang et al., 2011; Zhang Q. et al., 2015).

RNAi also provide unique opportunities in pest control (Toprak et al., 2013, 2014a). This strategy could include application (sprays) of single dsRNA or a combination of dsRNA with synergistics, chemicals, microbials or other molecules with insecticidal action. Many genes involved in lipid metabolism, such as *FAS*, *AKT*, *ACC*, and *CaM* have been targeted by RNAi for functional analysis, which resulted in impairment of lipid, carbohydrate and calcium metabolisms; as well as, inhibition of development, growth, reproduction, and even death (Cheon et al., 2006; Roy et al., 2007; Sim and Denlinger, 2009; Zhang Y. X. et al., 2015; Tan et al., 2017; Wang W. et al., 2018). Targeting peptide-hormones or their receptors, such as AKHR (Konuma et al., 2012; Alves-Bezerra et al., 2016; Hou et al., 2017), ILPs (Kim and Hong, 2015; Meng et al., 2015; Defferrari et al., 2016; Fu et al., 2016), sNPF (Dillen et al., 2013, 2014), NPF (Van Wielendaele et al., 2013), CrzR (Kubrak et al., 2016), and LkR (Yurgel et al., 2019) by RNAi also led to similar outcomes. Although the laboratory results are quite promising, transfer of this technology into field, in particular in relation to security for non-target organisms and financial cost, requires selection of specific genes and efficient dsRNA-synthesis technologies therefore further studies.

One alternative use of peptide hormones in pest management could be within the development of pest monitoring and forecasting strategies that are currently based on ecological parameters, primarily temperature. For example, AKH might be used as a marker for the prediction of emergence time of pests from hibernation, which would be also important to estimate migration times of pests from overwintering sites into the field in spring. Thus, AKH levels are elevated toward the end of hibernation in order to mobilize lipids prior to migration. Another one could be the DH which is also elevated by end of pupal diapause in *Heliothis* (Zhang et al., 2011). In sum, use of peptide hormones as biochemical markers of insect emergence and possibly epidemics is promising and worthed to focus; however, the concept is in the initial phase and requires further studies.

Overall, peptide-hormones have the potential for use in pest management, in particular with biological or chemical insecticides; however, the difficulty and the cost of peptide synthesis, the need for extensive (field-scale) amounts for application, as well as the necessity of techniques for efficient delivery are still drawbacks at this stage.

## Concluding Remarks

**FIGURE 5:**
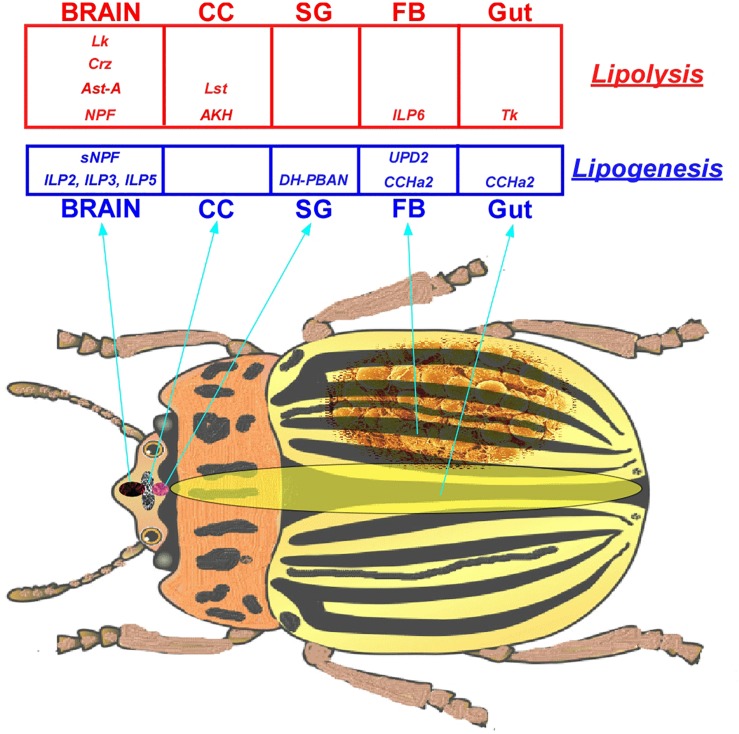
Lipoliytic and lipogenic peptide hormones and their synthesis site in an insect. Abbreviations: AKH, adipokinetic hormone; ILPs, insulin-like peptides, DH-PBAN, diapause hormone-pheromone biosynthesis activating neuropeptide; sNPF, short neuropeptide F; NPF, neuropeptide F; AstA, allatostatin-A; Crz, corazonin, Lk, leucokinin; CCHA2, CCHamide-2; Tk, tachykinins; Upd2, unpaired 2; Lst, limostatin.

Lipid metabolism is an ancient pathway with various common genetic actors and/or functional homologs from microorganisms to mammals. Insects have great potential to study lipid metabolism in related human disorders as they also share many common pathways. The fat body adipocytes and the hepatocyte-analogous oenocytes harbor events related to lipid metabolism, which are controlled through differential gene expression by transcription factors, post-transcriptional modifications, secondary messengers and hormones. Various peptide hormones, including neuropeptides, have many different effects on lipid metabolism through various pathways. One could say that the two most important groups of peptide hormones affecting lipid metabolism are AKH and the brain ILPs (ILP2, ILP3, and ILP5), the former induce lipolysis and the latter induce lipogenesis. Notably, the IGF-like ILP6 suppresses the production of brain ILPs and, therefore, might contribute into lipolysis. AstA, Crz, Lk, CCHa2, Tks, Lst, UPD1/2 and AdipoR affect lipid metabolism via their modulation on insulin secretion. NPF and sNPF are primarily involved in feeding behavior, therefore, affect lipid metabolism. Overall, AKH, ILP6, NPF, AstA, Crz, Lk Tk, and Lst stimulate lipolysis, while ILP2, ILP3, ILP5, DH-PBAN, sNPF, CCHa2, UPD1, and UPD2 induce lipogenesis (Figure 5). Although peptide hormones have diverse roles, they are also involved in other events, such reproduction, flight, diapause, starvation, and immunity, which are related to lipid metabolism. Finally, peptide hormones have promising potential to be used in pest control, in particular with biological or chemical insecticides; however, further studies are required in order to carry the approach into field.

## Author Contributions

UT conceptualized the study and wrote the manuscript.

## Conflict of Interest

The authors declare that the research was conducted in the absence of any commercial or financial relationships that could be construed as a potential conflict of interest.

## Abbreviations

ACBP1: Acyl-CoA-Binding Protein 1
ACC: Acetyl-CoA Carboxylase
ACP: AKH/Corazonin-related Peptide
Adipo: Adiponectin
AdipoR: Adiponectin Receptor
AGPAT3: 1-Acylglycerol-3-Phosphate Acyltransferase 3
AKH: Adipokinetic Hormone
AKHR: Adipokinetic Hormone Receptor
AKT: Serine-Threonine Protein Kinase
AMPK: AMP-activated Protein Kinase
APRP: AKH Precursor-Related Peptide
AstA: Allatostatin-A
ATGL: Adipose Triglyceride Lipase
Bmm: Brummer
CaM: Calmodulin
CaMKII: Calcium/Calmodulin-dependent Protein Kinase-II
cAMP: cyclic Adenosine monophosphate
CaN: Calcineurin
CCHa2: CCHamide-2
CCHa2R: CCHamide-2 Receptor
CREB: cAMP Response Element-Binding Protein
CRTC: cAMP-Regulated Transcriptional Co-activator
Crz: Corazonin
CrzR: Corazonin Receptor
DAG: Diacylglycerol
DAR2: Drosophila Allatostatin Receptor 2
DGAT1: Diacylglycerol O-Acyltransferase 1
DH: Diapause Hormone
DHR: Diapause Hormone Receptor
DILP: Drosophila Insulin-like Peptide
DOR: Diabetes and Obesity Regulated
DYRK1A: Dual − specificity Tyrosine Phosphorylation − regulated Kinase 1A
EcR: Ecdysone Receptor
ERK1: Extracellular signal-Regulated Kinase 1
ERK2: Extracellular signal-Regulated Kinase 2
E75: Ecdysone-induced Protein 75
4EBP: 4E Binding Protein
20E: 20-Hydroxyecdysone
FAS: Fatty Acid Synthase
FoxO: Forkhead Box Class O
FTZ-F1: Fushi Tarazu Factor 1
GABA: Gamma-aminobutyric acid
GPAT1: Glycerol-3-Phosphate-O-Acyltransferase 1
GPAT4: Glycerol-3-Phosphate-O-Acyltransferase 4
GPCR: G Protein-Coupled Receptor
G α q: G Protein α q Subunit
G γ 1: G Protein γ 1 Subunit
HDAC4: Histone Deacetylase 4
HR3: Hormone Receptor 3
HSL: Hormone Sensitive Lipase
IGF: Insulin-like growth-factor
IGFBP: Insulin Growth- Factor Binding Protein
ImpL2: Imaginal Morphogenesis Protein-Late 2
ILP: Insulin-like Peptide
InR: Insulin Receptor
IPBP: Insulin-related Peptide Binding Protein
IPC: Insulin producing cell
IP_3_: Inositol-1,4,5-trisphosphate
IP_3_R: IP_3_ Receptor
JH: Juvenile hormone
JHAMT: Juvenile Hormone Acid Methyltransferase
JNK: Jun N-Terminal Kinase
Kr-h1: Kruppel Homolog 1
LD: Lipid droplet
Lk: Leucokinin
LKB1: Liver Kinase B1
LkR: Leucokinin Receptor
LSD1: Lipid Storage Droplet-1
Lst: Limostatin
Mdy: Midway
miRNA: MicroRNA
Mnb: Minibrain
NPF: Neuropeptide F
Orai1: Plasma Membrane Calcium Channel Protein 1
PAP: Phosphatidic Acid Phosphatase
PBAN: Pheromone Biosynthesis Activating Neuropeptide
PEPCK: Phosphoenolpyruvate Carboxykinase
PI_3_K: Phosphoinositide 3 − Kinase
PIP_2_: Phosphatidylinositol bisphosphate
PIP_3_: Phosphatidylinositol trisphosphate
PKA: Protein Kinase A
PKB: Protein Kinase B
PKC: Protein Kinase C
PLC: Phospholipase C
PLIN 1: Perilipin 1
PTEN: Phosphatidylinositol-3,4,5-Trisphosphate 3-Phosphatase
RNAi: RNA interference
SERCA: Sarco/Endoplasmic Reticulum Calcium-ATPase
SIK: Salt-inducible kinase
SIK2: Salt-Inducible Kinase 2
SIK3: Salt-Inducible Kinase 3
sNPF: Short Neuropeptide F
sNPFR: Short Neuropeptide F Receptor
SOCE: Store-operated calcium entry
SREBP: Sterol Regulatory Element-Binding Protein
STIM: Stromal Interaction Molecule
TAG: Triacylglycerol
TGL: Triglyceride Lipase
Tk: Tachykinin
TkR: Tachykinin Receptor
TOR: Target of Rapamycin
TORC1: Target of Rapamycin Complex 1
UPD1: Unpaired 1
UPD2: Unpaired 2.
